# Highly Selective Electrolytic Reduction of CO_2_ to Ethylene

**DOI:** 10.1021/acsaem.5c01866

**Published:** 2025-09-03

**Authors:** Monsuru Olatunji Dauda, Mustapha Bello, John Hendershot, Nkechi Kingsley, Ignace Agbadan, Junghyun Park, Soundarzo Tasnim, Omotolani Oduyebo, Anthony Christian Engler, Craig Plaisance, John C. Flake

**Affiliations:** Cain Department of Chemical Engineering, Baton Rouge, Louisiana 70803, United States

**Keywords:** CO_2_ electroreduction, ethylene selectivity, pH optimization, copper-based electrocatalyst, membrane electrode assembly

## Abstract

We investigate the reduction of CO_2_ to ethylene across buffered anolyte pH values 4 to 14 using a copper–phosphorus (Cu–P) electrocatalyst in a zero-gap membrane electrode assembly. Electrochemical CO_2_ reduction using alkaline electrolytes typically shows limited carbon efficiencies and single-pass efficiencies, while acidic conditions typically favor the hydrogen evolution reaction. Results from this work show that weakly phosphate-buffered acidic anolytes (pH 6) maximize ethylene production with a 73% FE at 300 mA cm^–2^ and 51% FE at 500 mA cm^–2^, including a 51% single-pass CO_2_ conversion efficiency for over 400 h of continuous operation. We propose a mechanism based on pH-dependent CO coverage that controls the selectivity at the *HCCOH intermediate. Low CO coverage at pH 6 favors hydroxide elimination to *CCH, yielding ethylene (98% of C_2_ products), while high coverage at pH 14 promotes hydrogenation to ethanol (44% of C_2_). The HER mechanism transitions from H_2_O-mediated at pH 14 to phosphate-mediated (H_2_PO_4_^–^/HPO_4_^2–^) at weakly acidic pH, minimizing HER competition at pH 6. This mechanistic understanding enables controlled C_2_ product selectivity through manipulation of the CO coverage and local proton activity.

## Graphical abstract

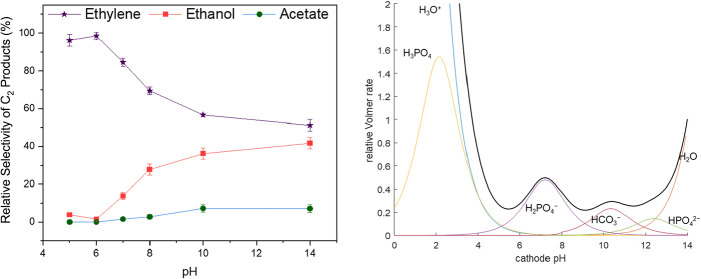

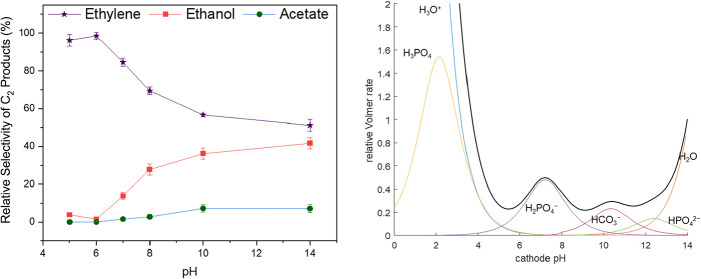

## 1 Introduction

Ethylene is the world’s highest production carbon-based chemical, with a per capita production of over 28 kg per person per year (globally exceeding 225 MMTA).^1,2^ It serves as the primary feedstock to produce hundreds of commercial products, including plastics, surfactants, lubricants, resins, fibers, and detergents.^2–4^ Current industrial production relies on pyrolytic cracking of fossil-based naphtha or ethane feeds at high temperatures (>750 °C). In addition to serving as the source of the feedstock, fossil fuels also typically supply heat for the reaction and energy for subsequent separations from byproducts. As a result, carbon intensities are typically reported near 1–2 kg of CO_2_-equivalent emissions per kilogram of fossil-based ethylene.^4–7^ In spite of this carbon intensity, ethylene is a basic building block, and its derivatives have tremendous advantages relative to their alternatives. For example, Life Cycle Analyses (LCA) studies addressing the recurring question of “paper versus plastic” tend to favor plastic from both an environmental impact and climate perspective.^8^ Thus, an efficient and selective means to produce ethylene using CO_2_, water, and renewable energy presents a promising path for carbon circularity.

The electrochemical conversion of CO_2_ to ethylene was first reported by Hori et al.^9^ in 1986 using Cu electrocatalysts in an H-cell configuration. Subsequent works using aqueous electrolytes and Cu electrocatalysts in H-cell configurations led to greater selectivity, albeit at low current densities, near 10 mA cm^–2^. Early works were limited in terms of pH because more acidic electrolytes favored the HER, and alkaline electrolytes favored carbonates (recognized as a dead end for CO_2_ reduction) and the relatively limited mass transfer rates of CO_2_. The introduction of GDEs in MEA cells in CO_2_ electrolysis by Kenis and colleagues^10^ allowed greater mass transfer, greater current densities (>100 mA cm^–2^), and the exploration of more alkaline environments that favored C–C coupling and suppression of the hydrogen evolution reaction (HER). However, the alkaline approach continues to face a fundamental limitation associated with carbonate formation via the chemical reaction of CO_2_ and OH^–^ and the establishment of a parasitic electrochemical pump.^11–16^ Notably, Kanan et al.^17^ showed that the single-pass efficiency is limited to 25% for ethylene production and CO_2_ pumping accounts for over 50% of the total system energy input.

CO_2_ reduction in acidic electrolytes mitigates carbonate formation and allows higher carbon efficiencies;^18–21^ however, competition with the HER remains a challenge. Huang et al.^19^ demonstrate a 77% single-pass CO_2_ conversion, including a 50% conversion to C_2+_ products, with an ethylene FE of approximately 31% at 1.2 A cm^–2^ in strongly acidic (pH < 1) phosphate electrolytes. The three-chamber flow cell operated at a cell potential of 4.2 V and demonstrated stable performance for 12 h using a novel cation-augmenting layer (CAL) incorporating a perfluorosulfonic acid (PFSA) ionomer. Similarly, Ma et al.^20^ demonstrated 84% FE for C_2+_ products (with ethylene contributing ∼40% FE) at 0.56 A cm^–2^ and a single-pass carbon efficiency of 54% for over 30 h using electrochemically reduced porous CuO nanosheet cathodes in a three-chamber flow cell (0.5 cm^2^ electrode). The acidic electrolyte (pH ≤ 1) included sulfuric acid (0.05 M H_2_SO_4_) with 3 M KCl. While these are significant improvements in CO_2_ reduction using relatively more acidic electrolytes (pH ≤ 1), however,^19,20^ because of the HER, the FE for ethylene was limited (below 50%)^20^ at current densities under 200 mA cm^–2^ for 30 h.^20^

Beyond the anolyte pH, cations are also known to play a role in the CO_2_ reduction activity and selectivity. Several studies have shown that high concentrations of alkali cations suppress the hydrogen evolution reaction (HER) and promote CO_2_ reduction (CO_2_R) by screening surface potential and reducing H^+^ transport.^22–24^ Proposed mechanisms include partially dehydrated cations facilitating CO_2_ adsorption through intermediate interactions and alkali ions at the outer Helmholtz plane, which help to reduce local electric fields. For example, Koper and colleagues^23^ considered CO_2_ reduction to CO at Au electrocatalysts in a two-compartment 10 cm^2^ GDE flow cell at a pH of 2–4 and demonstrated that weakly hydrated cations (K^+^ or Cs^+^) accumulate at the cathode and create a more alkaline local environment. Koper et al.^23^ also established a direct correlation between decreasing cation hydration strength and improved electrocatalytic performance (Li^+^ < Na^+^ < K^+^ < Cs^+^). The report^23^ included a CO_2_-to-CO FE of 80–90% at a current density of 200 mA cm^–2^ in sulfate-based electrolytes (pH 2–4) with 30% improved energy efficiency relative to neutral conditions (1 M KHCO_3_, pH = ∼8). Bell and colleagues^24^ further demonstrated that ethylene FEs increase in alkaline environments (1 M KHCO_3_, pH = ∼8) as cation size increases from a FE of 6.7% for Li^+^ to 9.3% for Na^+^, 31.5% for K^+^, 35.5% for Rb^+^, and ultimately reaching 39.6% with Cs^+^ at a cathode potential of −1 V vs RHE. This enhancement was attributed to the pH buffering effect of hydrated cations near the cathode surface, where the buffering capacity increases with cation size (Li^+^ < Na^+^ < K^+^ < Rb^+^ < Cs^+^). Larger cations result in lower local pH and higher CO_2_ concentrations near the electrode surface through cation hydrolysis, while also reducing the hydrogen evolution reaction through decreased polarization. These works suggest that similar cation behavior could be possible in weakly acidic electrolytes.

In this work, we investigate CO_2_ electroreduction from over a wide range of anolyte pH values (pH 4–14) using copper–phosphorus electrocatalysts in a MEA cell. We establish relationships between pH, product distribution, C_2_ selectivity, and CO_2_ conversion through single-pass carbon efficiency (SPCE) measurements. The effects of electrolyte composition are evaluated using alkali cations (Na^+^, K^+^, Cs^+^) and different anions, including PO_4_^3–^, NO_3_^–^, and SO_4_^2–^, to understand their roles in reaction pathways and product formation. Further, we explore the competition between CO_2_ reduction and the HER to understand reaction kinetics and rate-determining steps.

## 2 Experimental Section

### 2.1 Chemicals and Materials

The following methodologies described herein are patent pending. All solvents and chemical reagents were purchased from commercial sources and used as received without further purification. Copper­(II) chloride dihydrate (CuCl_2_·2H_2_O, ≥99%), hydrazine monohydrate (N_2_H_4_·H_2_O, 85%), sodium hypophosphite (NaH_2_PO_2_, 99%), iridium­(IV) chloride hydrate (IrCl_4_·*x*H_2_O, 99.9%), oxalic acid dihydrate (H_2_C_2_O_4_·2H_2_O, ≥99%), and hydrochloric acid (HCl, 37 wt %) were purchased from Thermo-Fisher Scientific. Alkali metal salts including potassium chloride (99.999% trace metals basis), lithium chloride (≥99.98% trace metals basis), cesium chloride (≥99.999% trace metals basis), and their corresponding hydroxides, potassium hydroxide (semiconductor grade, pellets, 99.99% trace metals basis), sodium hydroxide (NaOH, ≥98%), and cesium hydroxide (CsOH·H_2_O, 99.95%) were obtained from Sigma-Aldrich. The bicarbonates, including potassium bicarbonate (KHCO_3_, ≥99.99%) and cesium bicarbonate (CsHCO_3_, 99.9%), were sourced from Sinopharm Chemical Reagent Co., Ltd. Orthophosphoric acid (H_3_PO_4_, 85 wt% in H_2_O, 99.99% trace metals basis) was procured from Sigma-Aldrich. A Sustainion anion exchange membrane (X37–50 grade RT, dry thickness >50 μm), carbon black (Vulcan XC-72R), and titanium (Ti) felt were acquired from Fuel Cell Store. Research-grade CO_2_ (99.999%) and ultrahigh-purity argon were purchased from Airgas. Throughout all experiments, ultrapure water (18.2 MΩ·cm at 25 °C) was obtained from a Milli-Q water purification system.

### 2.2 Synthesis and Characterization

A one-pot approach was adapted from Dauda et al.^11^ to synthesize copper–phosphorus (Cu–P_0.065_) nanoparticles with a molar ratio of 1:0.065. Initially, 10 mmol of CuCl_2_·2H_2_O was dissolved in 50 mL of deionized water with vigorous stirring. The solution was stabilized with 1 g of poly­(vinylpyrrolidone) (PVP), and the pH was adjusted by gradually adding 12 M NaOH solution, forming a red liquid. The mixture was stirred at 80 °C for 2 h, and then 50 mL of a 1 mmol NaH_2_PO_2_ solution was added. Subsequently, 3 mL of N_2_H_4_·H_2_O was injected, and the mixture was heated and stirred for 3 h. The resulting Cu–P_0.065_ nanoparticles were collected by centrifugation, washed with deionized water, ethanol, and acetone, and dried at 60 °C for 12 h. Crystal and electronic structures of the samples were analyzed using X-ray diffraction (XRD, PANalytical) operated at 40 kV and 40 mA, with data collection from 20° to 100°. Surface morphology and elemental composition were examined by using scanning electron microscopy and energy-dispersive X-ray spectroscopy (SEM/EDS, Thermo Fisher PFIBSEM). X-ray photoelectron spectroscopy (XPS, Scienta Omicron) was used for the surface chemical state analysis.

### 2.3 Preparation of the Working Electrode

The working electrode preparation was adapted from previous work in our group.^11,25–30^ The procedure involved a spray-coating technique using a homogeneous electrocatalyst ink. The ink was prepared by first dispersing the Cu–P_0.065_ electrocatalyst (100 mg) in a mixture of deionized water (10 mL) and isopropanol (10 mL). To this suspension, 5 mg of conductive carbon (Vulcan Carbon) was added to enhance electrical conductivity, followed by 5.53 mg of polyvinylidene fluoride (PVDF) as a binding agent. The mixture was subjected to ultrasonication for 30 min to ensure uniform dispersion, followed by magnetic stirring for an additional 30 min to maintain suspension stability. The resulting homogeneous electrocatalyst ink was then spray-coated onto the microporous hydrophobic layer of a gas diffusion electrode (Sigracet 39BB, 50 cm^2^). The spraying was performed in multiple thin layers, allowing each layer to dry at room temperature before applying the next layer to ensure uniform electrocatalyst distribution. The electrocatalyst loading was carefully monitored by weighing the electrode before and after deposition using an analytical balance until achieving a final loading of 1.0 ± 0.1 mg cm^–2^.

### 2.4 Preparation of the IrO_2_/Ti-Mesh Anode

Building on established methods in our group,^11,25–30^ the iridium dioxide anode was prepared via a dip-coating and thermal decomposition method. First, titanium felt (6.64 cm^2^) underwent surface activation by etching in boiling 0.5 M oxalic acid solution for 30 min to remove surface oxides and enhance coating adhesion. The iridium precursor solution was prepared by dissolving IrCl_4_·*x*H_2_O (75 mg) in a mixture of 37% HCl (6.76 mL) and 2-propanol (18.24 mL). The activated Ti felt was then immersed in this solution, followed by a two-step thermal treatment: drying at 100 °C for 20 min and subsequent calcination at 500 °C for 20 min in air to convert IrCl_4_ to IrO_2_. This dip-coating and thermal decomposition cycle was repeated multiple times until achieving a uniform IrO_2_ loading of 3 mg cm^–2^, as determined by mass difference measurements.

### 2.5 Electrochemical Measurements

The electrochemical CO_2_ reduction reaction experiments were performed in a patented cell design. The cell architecture consisted of three main compartments: a cathode chamber, an anion exchange membrane (Sustainion), and an anode chamber. The cathode flow field was fabricated from 2205 stainless steel featuring a precise serpentine channel design (3.33 mm width, 0.2 mm depth) to optimize the CO_2_ gas distribution. Similarly, the anode flow field was precision-milled from grade 2 titanium with a serpentine channel configuration (0.79 mm width, 0.79 mm depth) for efficient electrolyte distribution. The cell employed an innovative design with independently adjustable clamping pressure on both anode and cathode end plates. This feature enabled precise control of compression force on the membrane electrode assembly, ensuring optimal contact between components while preventing membrane damage. The design eliminated the need for conventional gaskets and could accommodate variations in electrode thickness. Gas and electrolyte flows were precisely controlled throughout the experiments. High-purity CO_2_ (99.999%) was supplied to the cathode at a constant flow rate of 20 sccm, which was regulated by a mass flow controller (Alicat MC-500SCCM). The electrolyte composition was systematically varied using different concentrations of KOH (1.0 M) adjusted to various pH values (14–4) using H_3_PO_4_. A peristaltic pump (CHEM-TECH) maintained the electrolyte circulation at 1 mL min^–1^. Both gas and liquid flow rates were continuously monitored using Flow Vision 2.0 software to ensure stable operating conditions.

### 2.6 Product Analysis

Gas products from the electrochemical CO_2_ reduction were analyzed using a Shimadzu gas chromatograph (GC-2030) equipped with a 12 Stream Inlet Port and an autoinjector. The system incorporated both a thermal conductivity detector (TCD) and a flame ionization detector (FID) for comprehensive product analysis. Product separation was achieved using a Molecular Sieve 5A Capillary Column and a packed Carboxen-1000 Column with helium as the carrier gas. The FE for gaseous products was calculated according to

1 $${\mathrm{FE}}_{i}=x_{i}\times \frac{P_{o}V}{\mathrm{RT}}\times \frac{Z_{i}F}{I_{\mathrm{Total}}}\times 100\%$$

where *x*_*i*_ is the volume fraction of the gas product *i*, *V* is the outlet gas flow rate in m^3^ s^–1^, *P*_o_ is atmosphere pressure 101.325 kPa, *R* is the ideal gas constant 8.314 J mol^–1^ K^–1^, *T* is the room temperature in K, *Z_i_* is the number of electrons required to produce one molecule of product, *F* is the Faraday Constant 96,485 C mol^–1^, and *I*_Total_ is the total current in A.

Liquid products were collected from both electrode compartments using 10 mL of DI water in an ice bath (0 °C). Product quantification was performed using proton nuclear magnetic resonance spectroscopy (^1^H NMR) on an Agilent DD2 500 spectrometer, employing water suppression mode with dimethyl sulfoxide (DMSO) as the internal standard. Fresh anolyte was used for each 30 min collection period. The FE for liquid products was determined using

2 $${\mathrm{FE}}_{i}=n_{i}\times \frac{Z_{i}F}{I_{\mathrm{Total}}t}\times 100\%$$

where *n*_*i*_ is the quantity of the liquid product *i* in moles and *t* is the duration of product collection.

## 3 Results and Discussion

### 3.1 Electrocatalyst Synthesis and Characterization

The copper–phosphorus (Cu–P) electrocatalysts were synthesized using a one-pot process with hydrazine as the reducing agent and poly­(vinylpyrrolidone) (PVP) as a capping agent. Scanning electron microscopy (SEM) analysis revealed that the Cu–P electrocatalyst had a spherical nanoparticle morphology with an average diameter of around 100 nm (Figure 1A). Energy-dispersive X-ray spectroscopy (EDS) elemental mapping confirmed the uniform distribution of both copper and phosphorus elements within the nanoparticles (Figure 1B–C). This suggests the uniform incorporation of phosphorus into the copper matrix during the synthesis process. X-ray diffraction (XRD) analysis of the Cu–P electrocatalyst provided insights into its structural properties (Figure 1D). The characteristic XRD peaks correspond to the face-centered cubic Cu crystal structure. The Cu peaks were shifted to lower 2θ angles compared to undoped copper, indicating an expansion of the Cu crystal lattice, attributed to the incorporation of phosphorus into the Cu crystal structure. X-ray photoelectron spectroscopy (XPS) was used to investigate the electronic properties of the Cu–P electrocatalyst. The Cu 2p core level spectrum showed peaks at around 932 eV, corresponding to Cu^0^/Cu^+^ states, and a shoulder peak at 934 eV, associated with Cu^2+^ oxidation states (Figure 1E). The P 2p XPS spectrum (Figure 1F) indicated Cu–P or P–O species. The P 2p XPS spectrum peak at around 130 eV suggests a distinct Cu–P compound within the electrocatalyst.

**Figure 1:**
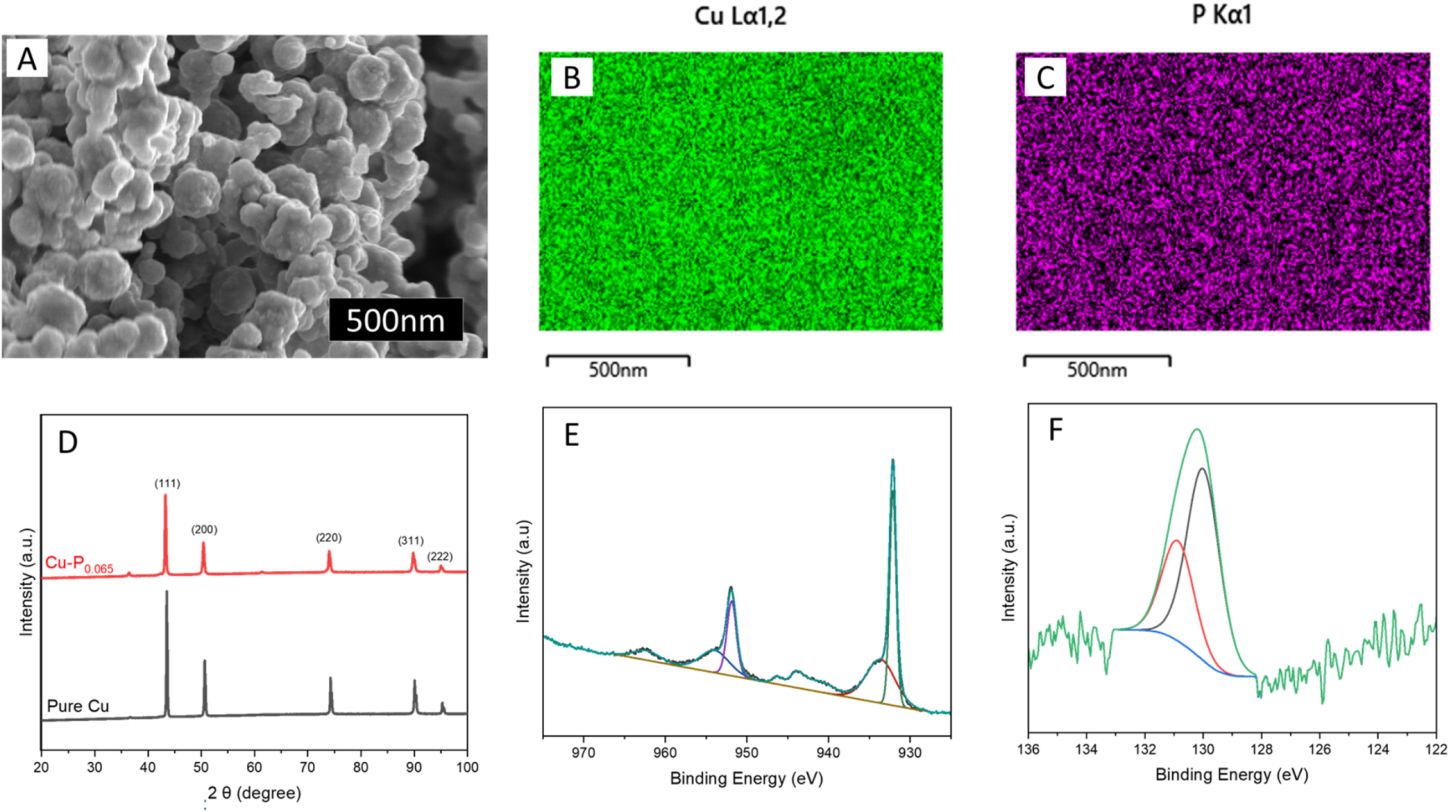
Morphology and structural characterization. The (A) SEM image of Cu–P showing spherical nanoparticles with average diameters around 100 nm. (B, C) EDS elemental mapping shows a uniform distribution of Cu and P elements. (D) XRD patterns of Cu–P, (E, F) Cu 2p, and P 2p XPS spectra of Cu–P.

### 3.2 Electrocatalyst and pH Effects on Product Distribution and Conversion

Copper–phosphorus (Cu–P) electrocatalysts were chosen for this study based on our previous work,^11,26,27,31^ which demonstrated that electronegative dopants can induce a partial positive charge (δ^+^) at Cu sites. This Cu^δ+^ character enhances the selectivity toward C_2_ products, with the Cu–P_0.065_ electrocatalyst (Cu^δ+^ = +0.13) exhibiting a 1.4-fold increase in ethylene FE compared to undoped Cu electrocatalysts. In contrast, electrocatalysts with higher partial positive Cu^δ+^ (e.g., Cu–Sn with +0.27, Cu_2_Se with +0.47) favor the production of oxygenates like ethanol and acetate over ethylene.^11,25^ The optimal 6.5% phosphorus content was determined through the investigation of Cu–P electrocatalysts with varying P/Cu ratios synthesized via chemical precipitation using hydrazine hydrate and NaH_2_PO_2_. Bulk and surface compositions of 2.8%, 6.5%, 7.7%, and 10.1% were confirmed by ICP-OES and XPS analyses, respectively (Table S1).

CO_2_ reduction was carried out on the membrane electrode assembly system depicted in Figure 2A, and we compare these results with previous CO_2_-to-ethylene reports^11,13,20,32–42^ in Figure 2B and Table S2. The membrane electrode assembly system featured a three-compartment design with an imidazolium-based AEM (Sustainion) separator and an independently adjustable clamping pressure mechanism. The system operated at a cathode flow rate of 20 sccm CO_2_ and anolyte flow rate of 1 mL min^–1^. Results using a constant cell potential of 4 V in 0.1 M KHCO_3_ (pH = 8) showed that Cu–P_0.065_ achieved the greatest C_2+_ selectivity with 81% total FE (51% ethylene, 25% ethanol, 3% acetate, 2% propanol), while suppressing C_1_ products to only 4% compared to 22% for undoped Cu. Electrocatalysts with lower P-contents (2.8%) resulted in reduced generation of C_2_ products, while higher doping levels (7.7% and 10.1%) (Figure 3A) resulted in increased hydrogen evolution reaction (HER) rates.^25,43^

**Figure 2:**
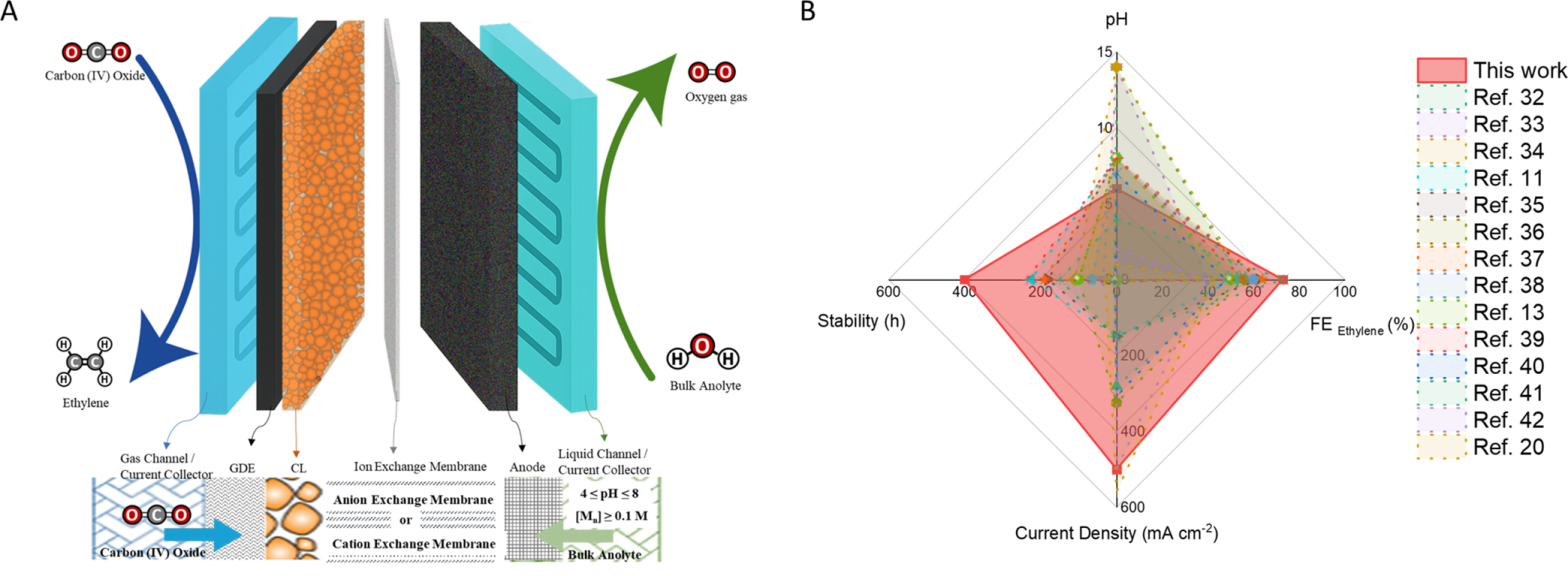
CO_2_ electrolysis to ethylene. (A) Schematic representation of the membrane electrode assembly system studied in this work. (B) Comparison of reported CO_2_-to-ethylene electrocatalytic systems.

**Figure 3:**
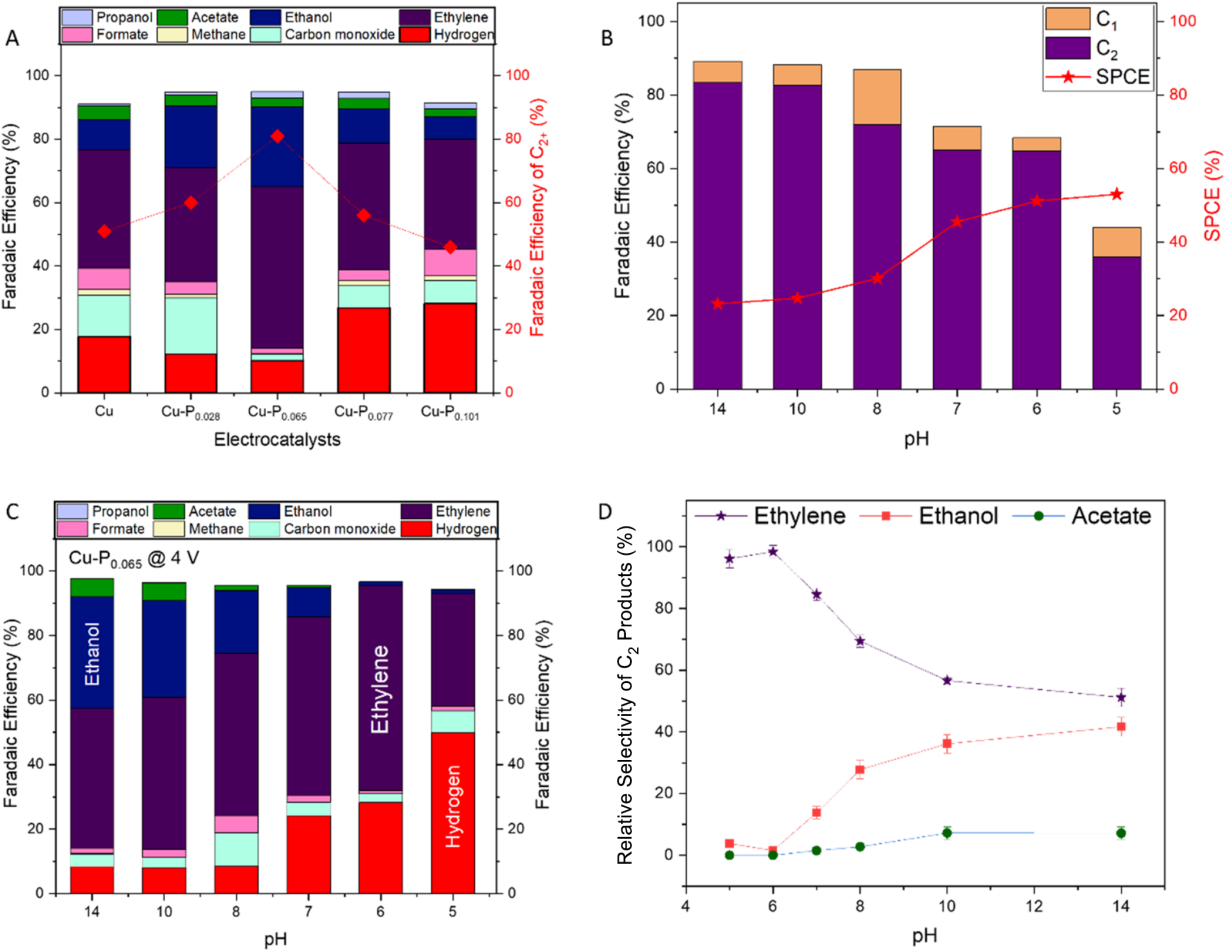
Electrocatalytic CO_2_ reduction performance at constant cell potential of 4 V. (A) The effect of phosphorus content on the FE distribution of CO_2_ reduction products in KHCO_3_ (pH = 8). (B) The influence of pH on the single-pass CO_2_ conversion efficiency (SPCE), C_1_ product selectivity, and C_2_ product selectivity during CO_2_ reduction on Cu–P_0.065_. (C) The effect of pH on the FE for various CO_2_ reduction products. (D) Relative selectivity distribution of C_2_ products across the pH range.

The role of phosphorus in Cu–P electrocatalysts involves serving as an electron donor to create partially positive copper sites (Cu^δ+^) that enhance catalytic performance.^11^ This electronic modulation occurs through electron donor–acceptor interactions based on phosphorus content, where experimental results and DFT calculations demonstrate that the Cu^δ+^ moiety facilitates the adsorption of carbon intermediates, C–C coupling, and promotes C_2_H_4_ generation energetically.^11,44^ The 6.5% phosphorus content represents optimal electronic modification without compromising copper activity, creating selective reaction pathways while the favorable Cu^δ+^ is reserved during CO_2_ reduction, contributing to long-term stability^11,44^ by preventing complete copper reduction and maintaining consistent performance over extended periods.

The potential for phosphorus leaching leading to structural reconstruction of the electrocatalyst during CO_2_ reduction, as reported in previous studies,^33^ was investigated through XPS analysis of the GDE before and after over 400 h of electrochemical testing. The survey spectrum analysis (Figure S1) reveals compositional changes where the phosphorus content decreases from 2.83% to 1.56% and the copper content decreases from 43.86% to 23.86%, while the carbon content increases correspondingly from 53.21% to 74.58%. The increase in carbon content is attributed to exposure of the underlying carbon gas diffusion electrode (GDE) support as Cu–P catalyst particles detach from the electrode surface during extended operation. However, the Cu:P ratio remains approximately constant at ∼15.5:1 before electrolysis and ∼15.3:1 after electrolysis, corresponding to the initial 6.5% phosphorus doping level. This consistent stoichiometry strongly indicates that phosphorus is not selectively leaching from the electrocatalyst structure, but rather, both copper and phosphorus are being lost proportionally due to physical detachment of electrocatalyst particles from the GDE support during CO_2_ reduction operation. The Cu LMM Auger analysis (Figure S2) comparing before (CuP-BE) and after (CuP-AE) CO_2_ reduction confirms that Cu­(I) remains the dominant oxidation state throughout the reaction period, while P 2p spectra (Figures S3–S4) show diminished but still detectable phosphorus signals after electrolysis, with clear P 2p_3/2_ and P 2p_1/2_ peaks visible before reaction (Figure S3) and reduced but persistent phosphorus presence after extended operation (Figure S4). The preservation of Cu/P stoichiometry, combined with the maintained copper oxidation state and detectable phosphorus presence after extended operation, demonstrates that the Cu–P electrocatalyst retains its structural integrity during CO_2_ reduction, with performance changes primarily attributed to overall electrocatalyst loading reduction rather than compositional alteration of the active phase.

CO_2_ reduction experiments were conducted using this copper–phosphorus (Cu–P_0.065_) electrocatalyst across a range of alkaline and acidic pH conditions (pH 14–4) using 1 M KOH (pH adjusted with phosphoric acid) in a zero-gap membrane electrode assembly (MEA) electrolyzer at constant cell potential of 4 V. In highly alkaline conditions (pH of 14), the FE for C_2_ products reached 83% (Figure 3B). This selectivity is attributed to the OH^–^ promotion of C–C coupling, as noted in the DFT calculations by Dinh et al.,^45^ which revealed that higher OH^–^ concentrations weaken surface binding and lower CO–CO coupling barriers. However, as the pH was decreased (more acidic), the C_2_ product selectivity declined, dropping to 65% at pH 6 and further reducing to 36% at the most acidic pH of 5. In contrast, the single-pass CO_2_ conversion efficiency (SPCE) exhibited the opposite trend. The SPCE was limited to 23% at pH 14 but increased to 25% at pH 10 and reached 54% at pH 5. This indicates that acidic conditions offer more efficient CO_2_ conversions. As the pH was further decreased, the FE for H_2_ production increased, from 8% at pH 14 to 19% at pH 5 (Figure 3C).

It is worth noting that while alkaline environments favor overall C_2_ selectivity, the distribution of C_2_ products shows remarkable pH dependence (Figure 3D). In highly alkaline conditions (pH 14, 1 M KOH), the electrocatalyst exhibits a greater selectivity to oxygenates, with ethanol relative selectivity reaching 42% compared to just 4% at pH 5. This may be attributed to high OH^–^ concentration promoting *OH reactions with *CO–CO intermediates to form an acetyl intermediate (H_3_CCO).^11^ In contrast, neutral and acidic conditions demonstrate high selectivity to ethylene, reaching 63.6% FE at pH 6 (comprising over 98% of all C_2_ products at this pH). Weakly acidic pH provides abundant surface protons for hydrogenation of C–C coupled intermediates, while the moderate Cu^δ+^ character of Cu–P_0.065_ maintains sufficient *CO binding for dimerization without excessive OH^–^ interaction that would favor the oxygenate pathway. These conditions also minimize the HER relative to strongly acidic environments, where a high H^+^ concentration kinetically favors hydrogen evolution.

### 3.3 Membrane System Performance

We performed CO_2_ reduction experiments using commercial cation exchange membranes (CEM, Nafion) and anion exchange membranes (AEM, Sustainion). Initial experiments with CEM (Nafion) using phosphoric acid H_3_PO_4_ anolytes showed a dominant HER (>93% FE) across pH 2–6, indicating insufficient CO_2_ activation under strongly acidic conditions (Figure 4A). Although K^+^ addition improved performance (Figure 4B), strong acid anolytes (pH 3–4) still favored HER (79–81%) FE, while weakly acidic anolytes (pH 5 and 6) increased ethylene FE to 27% with reduced HER (51–57%). The AEM system demonstrated better performance, showing a 64% ethylene FE with HER FE near 29% at pH 6 and maintaining stability over 400 h (Figure 4C). The performance difference between AEM and CEM systems is attributed to proton crossover in CEM systems that promotes HER, while AEM systems maintain optimal local pH conditions for CO_2_ activation.^46^ During the initial 0–50 h, ethylene FEs were near 70% ± 2%, the FE for hydrogen was 13% ± 1% with a cell potential of 3.8 ± 0.1 V, and overall CO_2_ conversion of 54% ± 2%. From 50 to 150 h, the ethylene FE dropped to 66% ± 2%, the hydrogen FE increased to 18% ± 1%, the cell potential increased to 4.2 ± 0.1 V, and the CO_2_ conversion decreased to 46% ± 2%. Between 150 and 400 h, the ethylene FE continued to decrease to 62% ± 2%, the hydrogen FE increased to 22% ± 1%, the cell potential reached 4.7 ± 0.1 V, and the CO_2_ conversion dropped to 38% ± 2%. These durability results indicate a gradual decline in catalytic performance over time, consistent with catalyst loading reduction due to physical detachment rather than decomposition processes.

**Figure 4:**
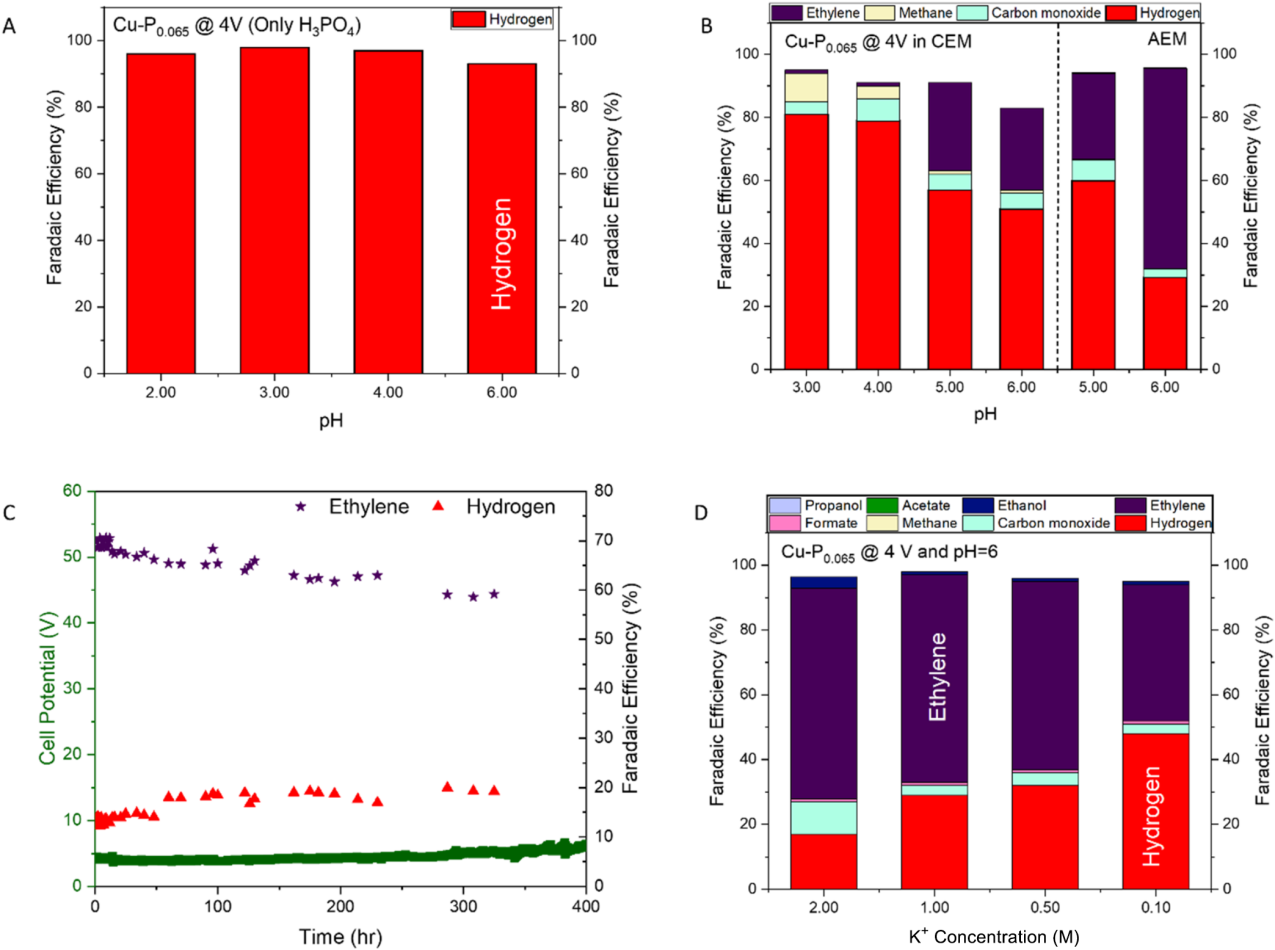
Membrane, pH, and cation effects on the CO_2_ electroreduction performance. (A) FE distribution across pH 2–6 in a CEM system using pure H_3_PO_4_. (B) FE distribution across different membranes after K^+^ (1 M) addition. (C) Long-term stability test of the AEM system at pH 6. (D) Effect of the K^+^ concentration (0.1–2 M) on the product distribution at pH 6.

Both membrane systems showed optimal ethylene production under weakly acidic conditions (pH 5–6). To understand the cation effect, we investigated the role of K^+^ at optimized weakly acidic conditions. The LSV curves in Figure S5 demonstrate that increasing K^+^ concentration from 0.5 to 3 M enhances catalytic activity, with current density rising from ∼35 mA cm^−2^ to ∼170 mA cm^−2^ at −1.5 V vs RHE. Higher K^+^ concentrations shift the onset potential positively and produce steeper curves, indicating improved reaction kinetics due to enhanced ionic conductivity and potential stabilization of reaction intermediates. Also, as shown in Figure 4D, increasing the K^+^ concentration from 0.1 to 2 M progressively suppressed H_2_ evolution from 48% to 20% FE while enhancing ethylene formation from 42% to 65% FE at pH 6 and 7 (Figure S6). Neutral anolytes without cations (DI water) showed HER as the dominant reaction (Figure S7).

The pH-dependent performance is believed to be governed by distinct HER mechanisms. In acidic media, the HER proceeds through direct proton reduction (H_3_O^+^ + e^–^ + *Cu → *Cu–H_ad_ + H_2_O), followed by the Heyrovsky pathway or Tafel recombination, with rapid kinetics impeding CO_2_ reduction. Weakly acidic conditions (pH 6) provide a moderate proton concentration that balances CO_2_ activation with reduced HER kinetics. Conversely, more alkaline conditions suffer from limited carbon utilization, high energy barriers for water splitting, which hinders surface hydrogen available for ethylene, and promotes oxygenates (ethanol and acetate).

### 3.4 Cation and Anion Effects on CO_2_ Reduction

We also investigated how electrolyte cations shape the CO_2_ electroreduction pathways through size and hydration properties. While ionic radii increase from Na^+^ (2.4 Å) to K^+^ (2.7 Å) to Cs^+^ (3.13 Å),^47^ hydration shell sizes decrease in reverse order. This affects product distribution, with larger cations suppressing the hydrogen evolution reaction (HER) from 31% (Na^+^) to 4% (Cs^+^), while enhancing C_2_ product formation from 45% to 89% FE at pH 14 (Figure 5A). Research by Liu et al.^48^ demonstrated that the driving force for OH desorption is larger for smaller cations (Na^+^) than larger cations (K^+^), explaining the HER activity trend of NaOH > KOH > CsOH. Further, Ringe et al.^49^ modeled how interfacial electric fields correlate with cation effects on CO_2_ reduction. Small cations (Na^+^) retain strong hydration shells, preventing electrode adsorption, while larger cations adsorb to the electrode surface, changing the outer Helmholtz plane potential. As large cations accumulate, electric field effects increase (Na^+^ to Cs^+^), stabilizing intermediates (*CO, *OCCO) for C_2_ products while destabilizing intermediates (*COOH, *CHOH) that produce C_1_ products. Product distribution results show that ethylene production increases from Na^+^ (25% FE) to K^+^ (43% FE), while ethanol production rises from 16% (Na^+^) to 39% (Cs^+^). The relative ethylene-to-ethanol ratio changes from 56:36 (Na^+^) to 47:44 (Cs^+^), showing that larger cations favor ethanol formation (Figure 5B). Also, near the electrode surface, larger cations undergo hydrolysis, creating a pH buffering system. This helps to maintain a near-neutral pH and promotes C_2_ products over the HER. Cs^+^ proves effective due to its size and hydration properties. Smaller cations favor C_1_ products (methane and CO), while large cations inhibit their formation, with formate production remaining low across all cations.

**Figure 5:**
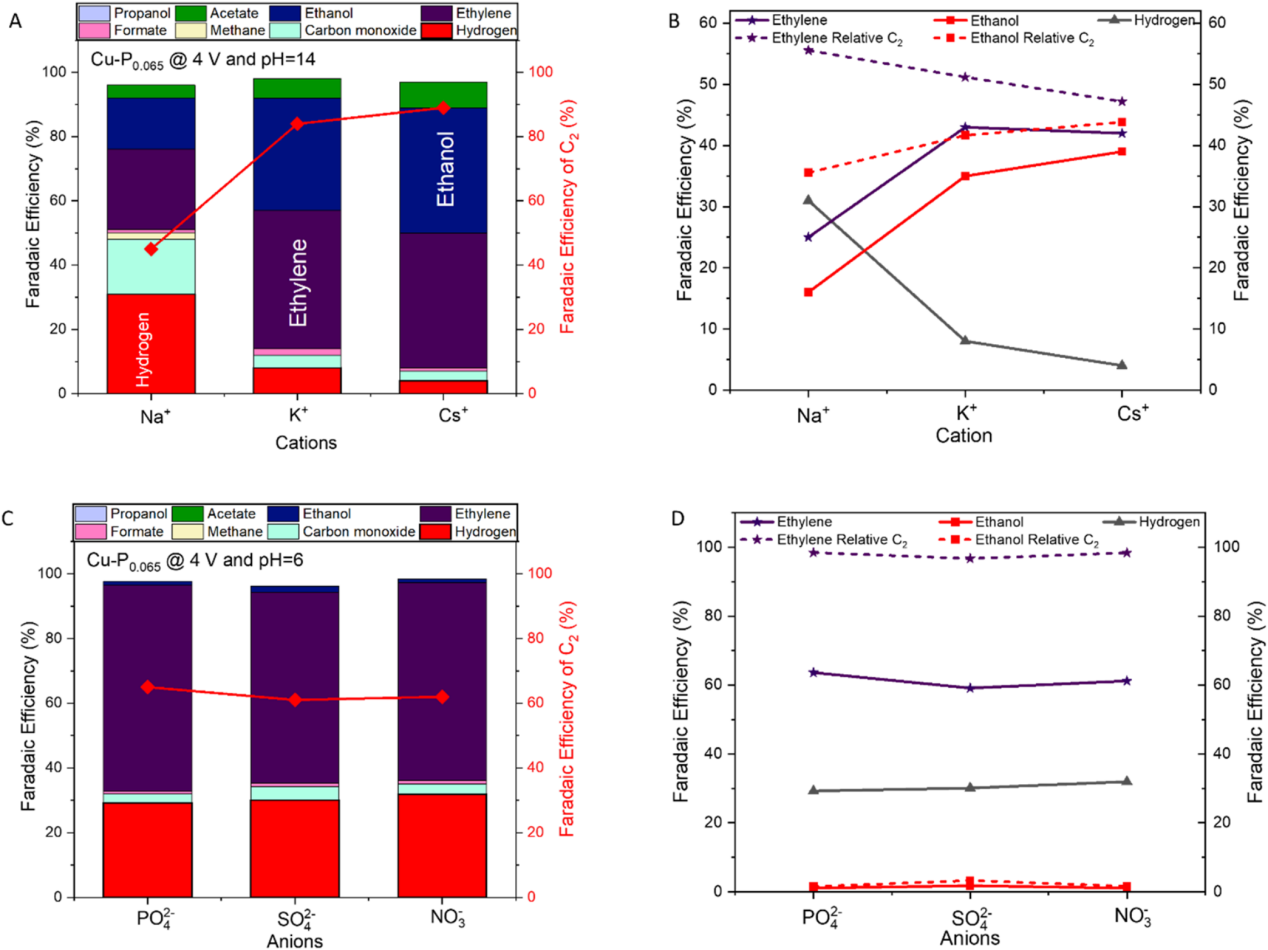
Effects of cation identity and anion type on CO_2_ electroreduction performance. (A) FE distribution across different cation systems (Na^+^, K^+^, and Cs^+^) at pH 14. (B) Relative selectivity of C_2_ products showing an ethylene:ethanol ratio at pH 14. (C) Faradaic efficiencies across different anions (PO_4_^2–^, SO_4_^2–^, and NO_3_^–^) at pH 6. (D) Relative selectivity of C_2_ products across anions at pH 6.

Also, while previous works^50–52^ have shown anions influence CO_2_ electroreduction through surface adsorption and local pH effects, and others have demonstrated anion-specific interactions with reaction intermediates, our research at fixed pH conveys that product distribution is primarily controlled by cation species and pH rather than anion effects. Studies with PO_4_^2–^, SO_4_^2–^, and NO_3_^–^ anions show consistent C_2_ product formation, with total C_2_ Faradaic efficiencies of 65%, 61%, and 62%, respectively (Figure 5C). The HER remains stable across anions (PO_4_^2–^: 29%, SO_4_^2–^: 30%, NO_3_^–^: 32%), unlike the significant variations observed with different cations (Na^+^: 31% to Cs^+^: 4%). Also, ethylene formation shows minimal variation across anions (PO_4_^2–^: 64%, SO_4_^2–^4:59%, and NO_3_^–^: 61%), contrasting with the marked cation effects. The relative selectivity between ethylene and ethanol remains consistent across anions, with ethylene dominating at 97–98% versus ethanol at 2–3% (Figure 4D). Other products like CO, CH_4_, formate, acetate, and propanol show negligible formation (0–4%) across all anions at pH 6, suggesting that anions may affect reaction kinetics but not the product distribution pathways.

### 3.5 Effect of Current Density on CO_2_ Reduction Performance

We investigated the influence of current density on the CO_2_ reduction performance using both Cu–P_0.065_ and unmodified Cu electrocatalysts across a range of current densities (100–500 mA cm^–2^). For both electrocatalysts, current density impacted total product distribution and selectivity between competing reaction pathways. For the Cu–P_0.065_ electrocatalyst at a low current density (100 mA cm^–2^), results showed high selectivity toward CO (46% FE) with moderate ethylene production (37% FE), indicating insufficient overpotential to efficiently couple CO intermediates into C_2_ products, while the hydrogen evolution reaction rate remained low (10% FE) under these conditions (Figure 6A). At 200 mA cm^–2^, the ethylene FE reached 67% while maintaining a low HER activity (8% FE). At 300 mA cm^–2^, the system reached maximum ethylene selectivity (73% FE) and minimal hydrogen evolution (8% FE). At 400 mA cm^–2^, the ethylene FE dropped to 64% while the HER FE increased to 30%. At 500 mA cm^–2^, performance further declined with the ethylene FE dropping to 51% while the HER FE increased to 37% FE.

**Figure 6:**
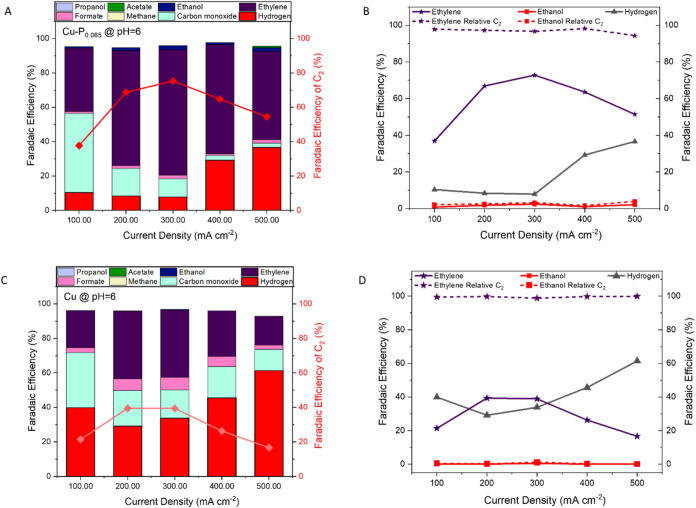
Effect of current density on CO_2_ reduction performance in the AEM system. (A) FE distribution for CO_2_ reduction products as a function of current density (100–500 mA cm^–2^) at pH 6 using Cu–P_0.065_ electrocatalyst. (B) Total C_2_ product selectivity and relative distribution of C_2_ products (ethylene and ethanol) across different current densities (100–500 mA cm^–2^) at pH 6 using the Cu–P_0.065_ electrocatalyst. (C) FE distribution for CO_2_ reduction products as a function of current density (100–500 mA cm^–2^) at pH 6 using an unmodified Cu electrocatalyst. (D) Total C_2_ product selectivity and relative distribution of C_2_ products (ethylene and ethanol) across different current densities (100–500 mA cm^–2^) at pH 6 using an unmodified Cu electrocatalyst.

This behavior indicates competing kinetic limitations: limited overpotentials to drive C_2_ formation at low current densities versus increased mass transport limitations, higher fields, and increased the HER at high currents. The relative selectivity between ethylene and ethanol remained in favor of ethylene (94–98%) across all current densities, suggesting that the reaction pathway is maintained at higher current densities (Figure 6B). Similar trends were observed at higher pH conditions, where Cu–P_0.065_ maintained high C_2_ selectivity but with different product distributions (Figures S8–S11). Other products, including methane, formate, acetate, and propanol, showed very low Faradaic Efficiencies (<2%) across all current densities.

The unmodified Cu electrocatalysts exhibited trends similar to those of Cu–P_0.065_ but with consistently lower Faradaic efficiencies for ethylene production across the current density range. At 100 mA cm^–2^, Cu showed moderate ethylene production (21% FE) but substantially higher hydrogen evolution (40% FE) and CO formation (32% FE) compared to Cu–P_0.065_ (Figure 6C). As current density increased to 200 mA cm^–2^, ethylene production improved to a maximum of 40% FE, while hydrogen evolution decreased to 29% FE and CO production declined to 21% FE. At 300 mA cm^–2^, ethylene production maintained a similar level, including a 39% FE, while hydrogen evolution slightly increased to 34% FE and CO continued to decrease (16% FE).

Further increasing the current density with unmodified Cu resulted in a more pronounced shift toward hydrogen evolution. At 400 mA cm^–2^, ethylene production declined substantially to 26% FE while hydrogen evolution increased significantly to 46% FE. This trend intensified at 500 mA cm^–2^, where ethylene FE dropped further to 17% FE and hydrogen production dominated the product distribution with 61% FE. The relative selectivity between ethylene and ethanol for Cu remained in favor of ethylene (>99%) across most current densities (Figure 6D). This behavior was consistent across different pH environments, as demonstrated in additional experiments at pH 8 and 14 (Figures S12–S15).

### 3.6 Nature of Selectivity

We propose the general mechanism depicted in Figure 7 to explain the increased selectivity for ethylene over ethanol at lower anolyte pH values. The cycle begins with the adsorption of CO_2_ on the surface to form a carboxylate intermediate, followed by protonation and hydroxide elimination to form CO. The adsorbed CO then dimerizes to give A1, which readily protonates to HOCCOH (A2). This intermediate subsequently undergoes hydroxide elimination to yield CCOH (A3) and protonation to form HCCOH (A4). The HCCOH intermediate can follow one of two pathways, the first being hydroxide elimination to form CCH (B1), followed by hydrogenation to ethylene. Alternatively, it can directly undergo a series of hydrogenation steps to yield ethanol, passing through protonated ethenone (A5) and acetyl (A6) intermediates. A third pathway branches off from the protonated ethenone, where it deprotonates and desorbs to eventually yield acetate. Although significant yields of acetate are not observed in this work, a parallel study performed in our group demonstrated that further increasing the CO coverage by cofeeding CO favors this pathway.

**Figure 7:**
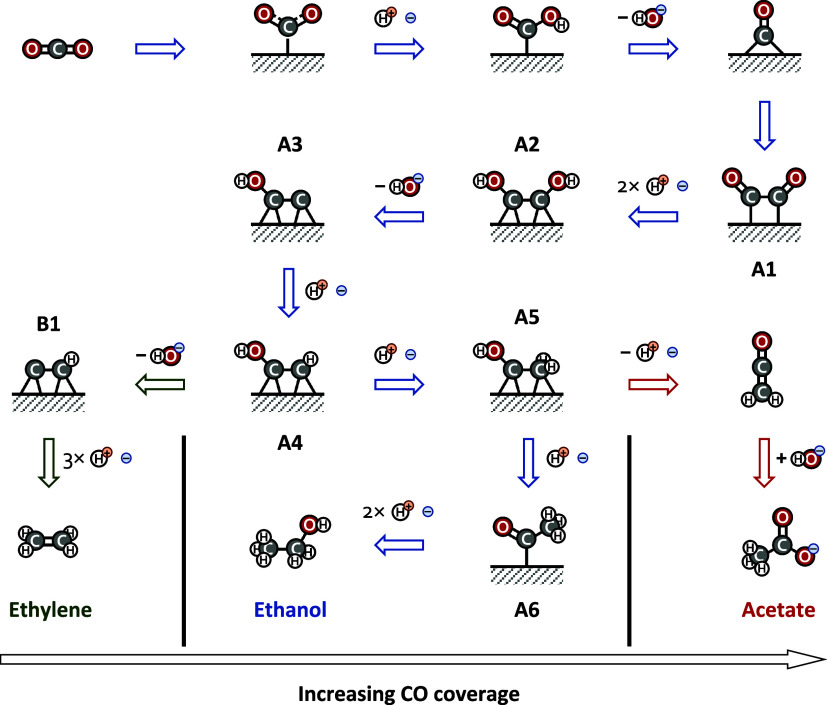
Proposed mechanism for the formation of C_2_ products from CO_2_ electrolysis. As CO coverage increases, selectivity shifts from ethylene to ethanol and then to acetate.

The shift in selectivity from ethylene to ethanol as the anolyte pH increases can be explained in terms of the resulting variation in the CO coverage. The steady state CO coverage is controlled by the relative rate coefficients for CO formation by CO_2_ activation versus CO consumption by dimerization. It has been widely reported that the rate-limiting step for CO_2_ activation is the initial adsorption on the surface to form the carboxylate intermediate.^53–57^ The rate of this step has been shown to increase as the electrolyte pH increases when the electrode potential is held constant with respect to the RHE (so that the potential increases on the SHE scale).^56^ This is explained in terms of the increase in the interfacial electric field stabilizing the partial negative charge on the carboxylate intermediate.^58^ On the other hand, the CO dimerization step is less sensitive to the pH (at constant RHE potential), leading to an increase in CO coverage at higher pH. A similar effect has been observed when the electrolyte contains a higher concentration of larger cations, whereby the CO coverage has been found to increase.^59^

As the CO coverage increases, repulsive interactions with surface intermediates weaken their binding to the surface, altering the relative barriers of the pathways branching out from the HCCOH (A4) intermediate. Hydrogenation of HCCOH leads to a more weakly binding H_2_CCOH (A5) intermediate, while the competing hydroxide elimination step leads to a more strongly binding CCH intermediate (B1). Consequently, the increased CO coverage at high pH and with larger cations present decreases the activation barrier for hydrogenation to form ethanol while increasing the activation barrier for the hydroxide elimination step to form ethylene. This explains the observed increase in selectivity to ethanol over ethylene as the pH increases, the cation concentration increases, or larger cations are present in the electrolyte.

Although the C_2_ product selectivity favors ethylene over ethanol as the pH decreases from 10 to 5, the competing HER also becomes more favorable at a lower pH. We rationalize this in terms of pH-induced variations in the concentrations of various proton donors in the electrolyte. As shown in Figure 8, water is the only proton donor present at high pH with a high concentration and necessarily must be the one participating in any transition state involving proton transfer to the surface, such as the Volmer and Heyrovsky steps of the HER. When pH decreases, more acidic proton donors like HPO_4_^2–^, HCO_3_^–^, and H_2_PO_4_^–^ increase in concentration and eventually replace water as the dominant proton donor. One would expect that more acidic proton donors can transfer a proton to the surface with lower kinetic barriers than less acidic proton donors, leading to an increase in the rate of the HER at lower pH. At the same time, the rate of CO_2_ activation decreases due to the correlation between the rate of CO_2_ adsorption and pH discussed earlier. This explains the selectivity increase for the HER relative to the CO_2_ reduction observed with decreasing pH.

**Figure 8:**
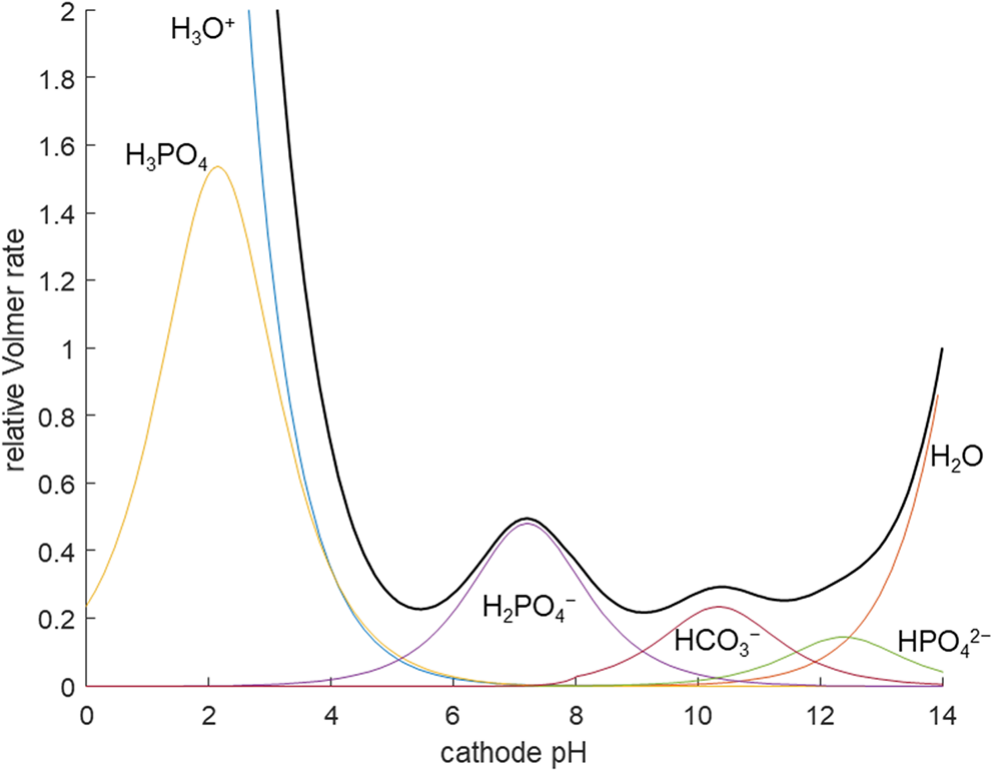
Relative rates of the Volmer step for five different proton donors as a function of electrolyte pH at a constant *U*_RHE_. The black line shows the total HER rate for all proton donors in an electrolyte with 1 mol/L total phosphate.

The effect of pH on HER activity is illustrated in Figure 8 using a simple model of HER kinetics based on a Marcus theory-based approach to activation barriers that we reported in previous work.^60^ The activation barrier Δ*G* ^‡^ is given by

3 $$\Delta G^{‡}=\frac{{\left(\Delta G-\beta_{\mathrm{HB}}+\beta_{B}+\lambda \right)}^{2}}{4\lambda }+\beta_{\mathrm{HB}}$$

where Δ*G* is the free energy of the Volmer step and λ is the reorganization energy, while the quantities β_HB_ and β_B_ account for the free energy penalty associated with moving the proton donor HB or its conjugate base B from the bulk electrolyte to the active site. The dependence of the free energy Δ*G* on the electrode potential and p*K*_a_ of the proton donor is given by

4 $$\Delta G=\Delta G_{0}+U_{\mathrm{SHE}}+0.059\times pK_{a}=\Delta G_{0}+U_{\mathrm{RHE}}+0.059\times \left(pK_{a}-\mathrm{pH}\right)$$

where Δ*G*_0_ is the value for a proton donor with a p*K*_a_ of 0 at a potential of zero on the SHE scale. We then propose that the reorganization energy has a linear dependence on the p*K*_a_ of the proton donor

5 $$\lambda =\lambda_{0}+4\times 0.059\times \alpha pK_{a}$$

so that it decreases for more acidic donors. Finally, the rate of the HER can be expressed as

6 $$r=\frac{k_{B}T}{h}\exp\left(-\frac{\Delta G^{‡}}{k_{B}T}\right)\left[\mathrm{HB}\right]$$

where [HB] is the concentration of the proton donor.

Figure 8 shows the variation in rate with pH at constant *U*_RHE_ for an electrolyte containing 1 mol/L total dissolved phosphate (PO_4_^3–^, HPO_4_^2–^, H_2_PO_4_^–^, H_3_PO_4_) and up to 1 mol/L total dissolved carbon (CO_3_^2–^, HCO_3_^–^, and H_2_CO_3_). Additionally, the total dissolved carbon is limited by the requirement that the partial pressure of CO_2_ in equilibrium with it must be less than 1 atm. The total rate is plotted along with the individual rates associated with each proton donor (H_2_O, HPO_4_^–^, H^2^PO_4_^2–^, H_3_PO_4_, HCO_3_^–^, and H_3_O^+^). The rate of each proton donor was computed according to eq 5 using parameters obtained from a DFT calculation of the Volmer step on Cu(100) with an H_2_O proton donor (details are given in the Supporting Information (SI) note). One can see that H_2_O is the dominant proton donor when the electrolyte pH is close to 14, with a rate that increases with increasing pH. This increase is due to the decrease in *U*_SHE_ with pH when *U*_RHE_ is held constant, which in turn leads to a higher cathodic electric field at the interface that drives the transfer of the positively charged proton from the electrolyte to the surface. Likewise, H_3_O^+^ is the dominant proton donor when the pH is close to 0, with a rate that increases with decreasing pH due to the associated increase in H_3_O^+^ concentration.

At moderate pH values, the rate of the Volmer step is dominated by phosphate-based proton donors. The individual rate associated with each of these donors exhibits a maximum when the pH is close to the donor p*K*_a_, which can be explained by opposing influences of the interfacial electric field and the proton donor concentration. As the pH decreases below the p*K*_a_ of the donor, the electric field at the interface becomes less cathodic, which leads to a reduction in driving force for transfer of the proton from the electrolyte to the surface. On the other hand, as the pH increases above the p*K*_a_ of the donor, its concentration rapidly decreases as the deprotonated form becomes thermodynamically favored over the protonated form. Even though the electric field is becoming more cathodic, the concentration effect is stronger, so that the rate decreases.

The total rate of the Volmer step exhibits a maximum at the acidic and alkaline ends of the pH range associated with the H_3_O^+^ and H_2_O proton donors, while also exhibiting a local maximum close to a pH of 7 that is associated with the H_2_PO_4_^–^ proton donor. The increase in HER selectivity observed in Figure 3C with decreasing pH can be explained by the increase in the rate associated with H_2_PO_4_^–^. This is the dominant proton donor at pH values between 7 and 10, and its concentration increases as the pH decreases in this range, leading to a higher rate for carrying out the Volmer step.

## 4 Conclusions

These results demonstrate that weakly acidic conditions (pH 6) provide an optimal environment for electrochemical CO_2_ reduction to ethylene at Cu–P electrocatalysts. By investigating CO_2_ reduction across a wide pH range (4–14) in a zero-gap membrane electrode assembly, we established that weakly acidic environments balance CO_2_ activation with controlled hydrogen evolution to achieve 73% FE for ethylene at 300 mA cm^–2^ and 51% FE at 500 mA cm^–2^ while enabling 51% single-pass CO_2_ conversion efficiency. The Cu–P_0.065_ electrocatalyst, characterized by optimal phosphorus doping concentration (6.5%), shows enhanced *CO generation and C–C coupling while maintaining remarkable stability over 400 h with ± 10% change in both ethylene FE and HER. A key insight is that CO coverage, which increases with pH, likely contributes to the competition between ethylene and ethanol formation pathways. At the *HCCOH intermediate, low CO coverage at pH 6 enables hydroxide elimination to form *CCH species, leading to ethylene (98% of C_2_ products), while high coverage at alkaline pH weakens surface binding, favoring hydrogenation to ethanol via *H_2_CCOH (44% of C_2_ at pH 14). Our kinetic model reveals pH-dependent proton donor transitions from H_2_O at pH 14 to phosphate species (H_2_PO_4_^–^/HPO_4_^2–^) at neutral pH, providing a balance between proton availability and HER suppression.

## Supplementary Material



## Notes

XPS characterization of Cu–P electrocatalyst before and after CO_2_ electroreduction; elemental composition analysis of Cu–P electrocatalysts (ICP-OES and XPS data); CO_2_ reduction LSV curves showing K^+^ concentration effects; Faradaic efficiency and product distribution data at various current densities (100–500 mA cm^–2^) and pH values (6, 7, 8, 14); comparative performance of Cu–P_0.065_ versus unmodified Cu catalysts; relative distribution of C_2_ products (ethylene and ethanol) across different operating conditions; and comparative table of MEA electrolyzer systems for ethylene production highlighting stability data; theory calculations examining the Volmer step with different proton donors, providing theoretical insights into the enhanced C_2_ product formation on phosphorus-modified copper surfaces (PDF)
