# Supplementary material for: Highly Selective Electrolytic Reduction of CO2 to Ethylene
Source: ACS Appl Energy Mater. 2025 Sep 3;8(18):13607–19. doi: 10.1021/acsaem.5c01866 (PMC12458464; doi:10.1021/acsaem.5c01866)
Supplement: Supplementary file 1 [file ae5c01866_si_001.pdf]

Supporting Information

Highly Selective Electrolytic Reduction of CO_2 to Ethylene

Monsuru Olatunji Dauda[1], Mustapha Bello[1], John Hendershot[1], Nkechi Kingsley[1], Ignace Agbadan[1], Junghyun Park[1], Soundarzo Tasnim[1], Omotolani Oduyebo[1], Anthony Christian Engler[1], Craig Plaisance[1], John C. Flake[1]*

[1]*Cain Department of Chemical Engineering, Baton Rouge, Louisiana 70803, USA.*

Corresponding Author
John C. Flake − Gordon A. and Mary Cain Department of Chemical Engineering, Louisiana State University, Baton Rouge, Louisiana 70803, United States.

Email: johnflake@lsu.edu

The PDF includes:

Table S1: Elemental composition analysis of Cu-P electrocatalysts. ICP-OES data showing bulk composition and XPS data showing surface composition (normalized to Cu and P only, excluding C and O contributions). 4

Figure S1. XPS characterization of Cu-P catalyst before and after CO_2 electroreduction. Survey spectra showing elemental composition changes, with tabulated atomic percentages for C 1s, P 2p, and Cu 2p before electrolysis (BE) and after electrolysis (AE). 5

Figure S2. XPS characterization of Cu-P catalyst before and after CO2 electroreduction. Cu LMM Auger spectra comparing Cu-P catalyst before (CuP-BE) and after (CuP-AE) CO_2 reduction, confirming dominant Cu(I) oxidation state preservation. 6

Figure S3. XPS characterization of Cu-P catalyst before and after CO2 electroreduction. High-resolution P 2p spectrum before electrolysis showing distinct P 2p3/2 and P 2p1/2 peaks. 7

Figure S4. XPS characterization of Cu-P catalyst before and after CO2 electroreduction. High-resolution P 2p spectrum after electrolysis demonstrating reduced but persistent phosphorus signals, indicating retained P-doped structure rather than selective leaching. 8

Figure S5: CO_2RR LSV curves of Cu-P showing the effect of K^+ concentration on catalyst performance in 0.5 M to 3.0 M KOH. 9

Figure S6. Effect of K+ concentration (0.1-2M) on product distribution at pH 6 10

Figure S7. Faradaic Efficiency (%) versus Current Density (mA cm^{-2}) for Cu-$P_{0.065}$ at pH=7 in DI water. 11

Figure S8. FEdistribution for CO_2 reduction products as a function of current density (100-500 mA cm^{-2}) at pH 14 using Cu-$P_{0.065}$ electrocatalyst in AEM system. 12

Figure S9. Relative distribution of C_2 products (ethylene and ethanol) across different current densities (100-500 mA cm^{-2}) at pH 8 using Cu-$P_{0.065}$ electrocatalyst. 13

Figure S10. FEdistribution for CO_2 reduction products as a function of current density (100-500 mA cm^{-2}) at pH 14 using Cu-$P_{0.065}$ electrocatalyst in AEM system. 14

Figure S11 Relative distribution of C_2 products (ethylene and ethanol) across different current densities (100-500 mA cm^{-2}) at pH 14 using Cu-$P_{0.065}$ electrocatalyst. 15

Figure S12. FEdistribution for CO_2 reduction products as a function of current density (100-500 mA cm^{-2}) at pH 8 using Cu electrocatalyst in AEM system. 16

Figure S13. Relative distribution of C_2 products (ethylene and ethanol) across different current densities (100-500 mA cm^{-2}) at pH 6 using Cu electrocatalyst. 17

Figure S14. FEdistribution for CO_2 reduction products as a function of current density (100-500 mA cm^{-2}) at pH 14 using Cu electrocatalyst in AEM system. 18

Figure S15. Relative distribution of C_2 products (ethylene and ethanol) across different current densities (100-500 mA cm^{-2}) at pH 14 using Cu electrocatalyst. 19

Table S2: Comparison to previous MEA electrolyzer for ethylene 20

Supporting Note: Density functional theory calculations 21

Figure S16. Optimized structures for the reacant precursor state, the transition state, and the product precursor state. 27
Table S3. Results of VASP/VASPsol++ calculations. All energies are in eV. 28
Table S4. Parameters for the kinetic model of the Volmer step in eV. 28
Table S5. pK_a values for the proton donors used in the kinetic model 29
References 31

Table S1: Elemental composition analysis of Cu-P electrocatalysts. ICP-OES data showing bulk composition and XPS data showing surface composition (normalized to Cu and P only, excluding C and O contributions).

Electrocatalyst	ICP-OES			XPS		
	Cu concentration (ppm)	P concentration (ppm)	P/Cu Ratio (%)	Cu (%)	P (%)	P/Cu Ratio (%)
**Cu**	56.25	-	-	100.00	-	-
**Cu-P$_{(0.028)}$**	52.17	1.46	2.80	97.30	2.70	2.77
**Cu-P$_{(0.065)}$**	43.82	2.85	6.50	93.90	6.10	6.50
**Cu-P$_{(0.077)}$**	46.85	3.61	7.71	92.90	7.10	7.64
**Cu-P$_{(0.101)}$**	39.88	4.03	10.11	90.90	9.10	10.01

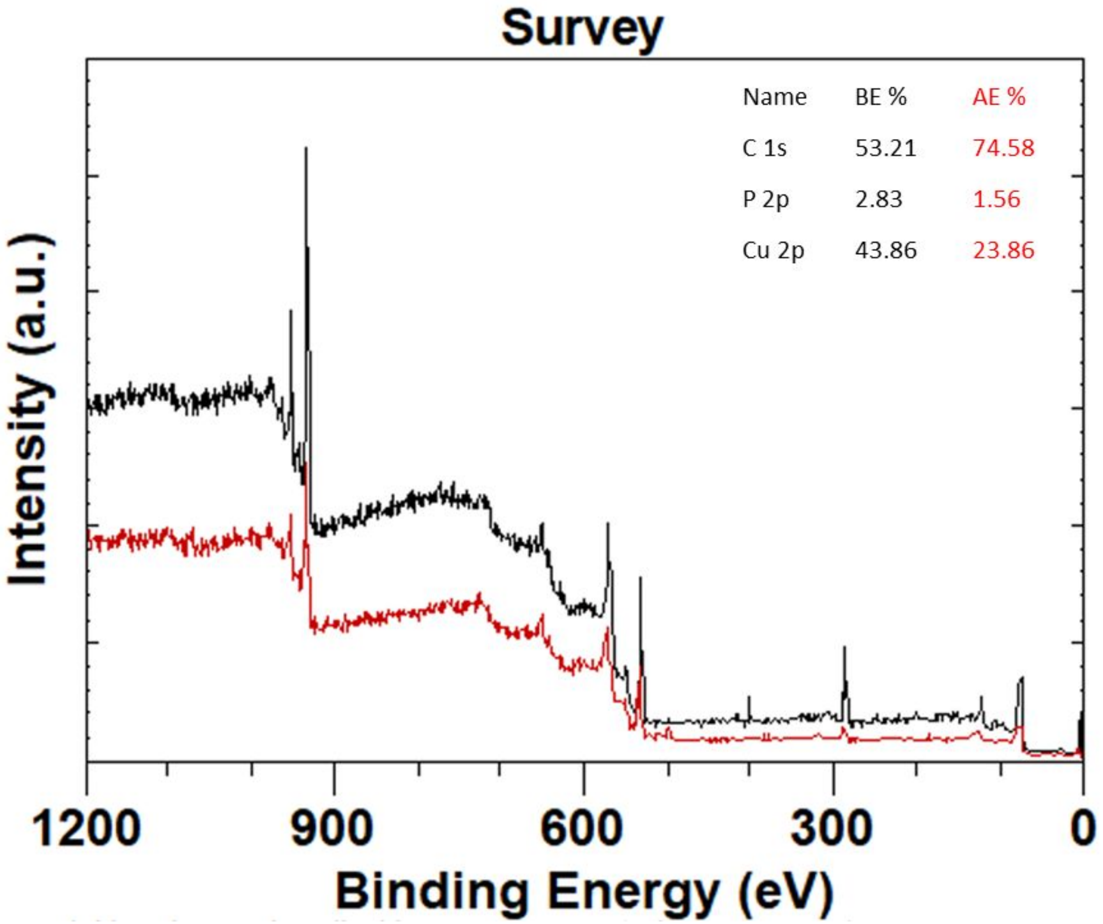

Figure S1. XPS characterization of Cu-P catalyst before and after CO_2 electroreduction. Survey spectra showing elemental composition changes, with tabulated atomic percentages for C 1s, P 2p, and Cu 2p before electrolysis (BE) and after electrolysis (AE).

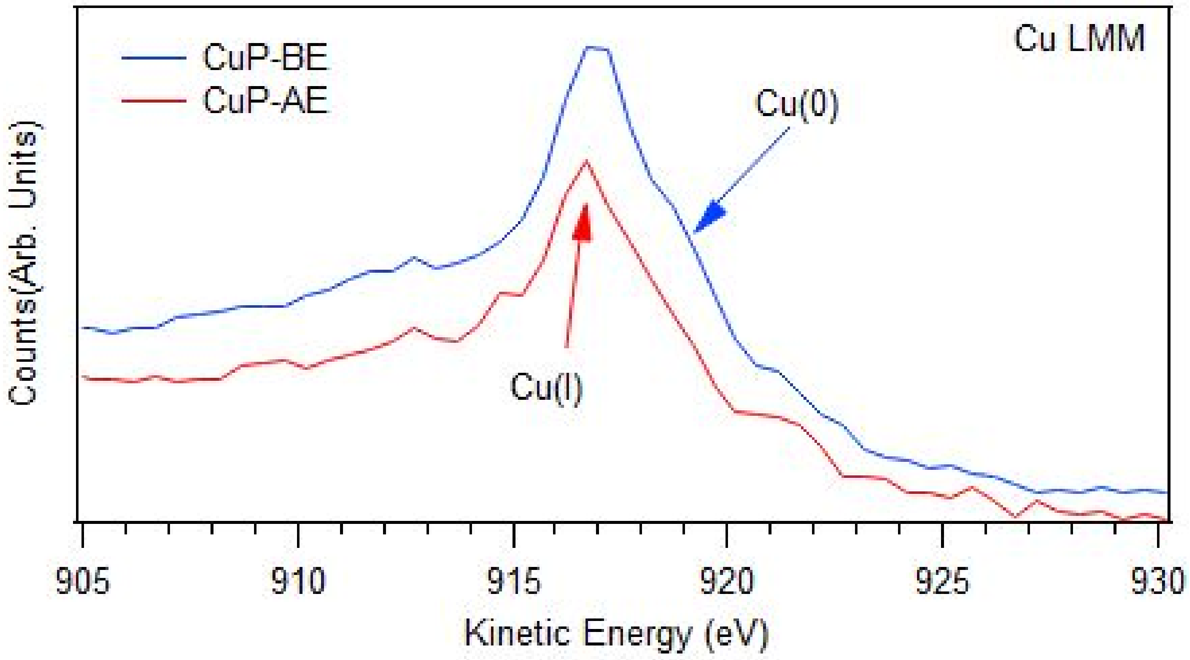

Figure S2. XPS characterization of Cu-P catalyst before and after CO2 electroreduction. Cu LMM Auger spectra comparing Cu-P catalyst before (CuP-BE) and after (CuP-AE) CO_2 reduction, confirming dominant Cu(I) oxidation state preservation.

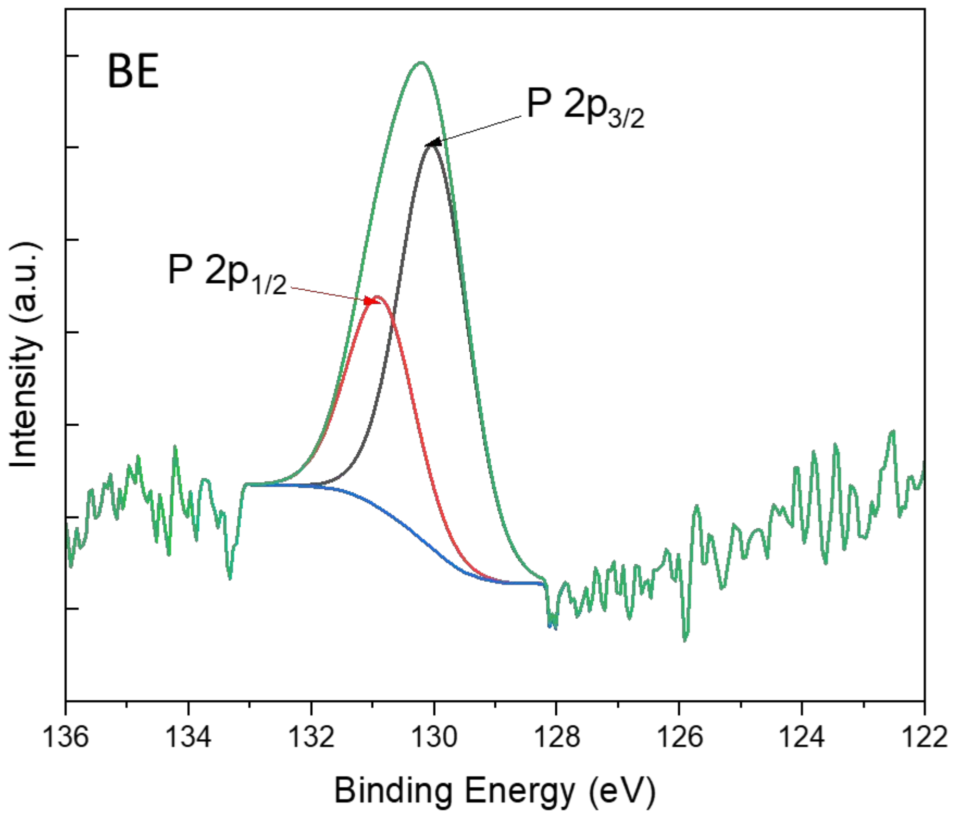

Figure S3. XPS characterization of Cu-P catalyst before and after CO2 electroreduction. High-resolution P 2p spectrum before electrolysis showing distinct P 2p3/2 and P 2p1/2 peaks.

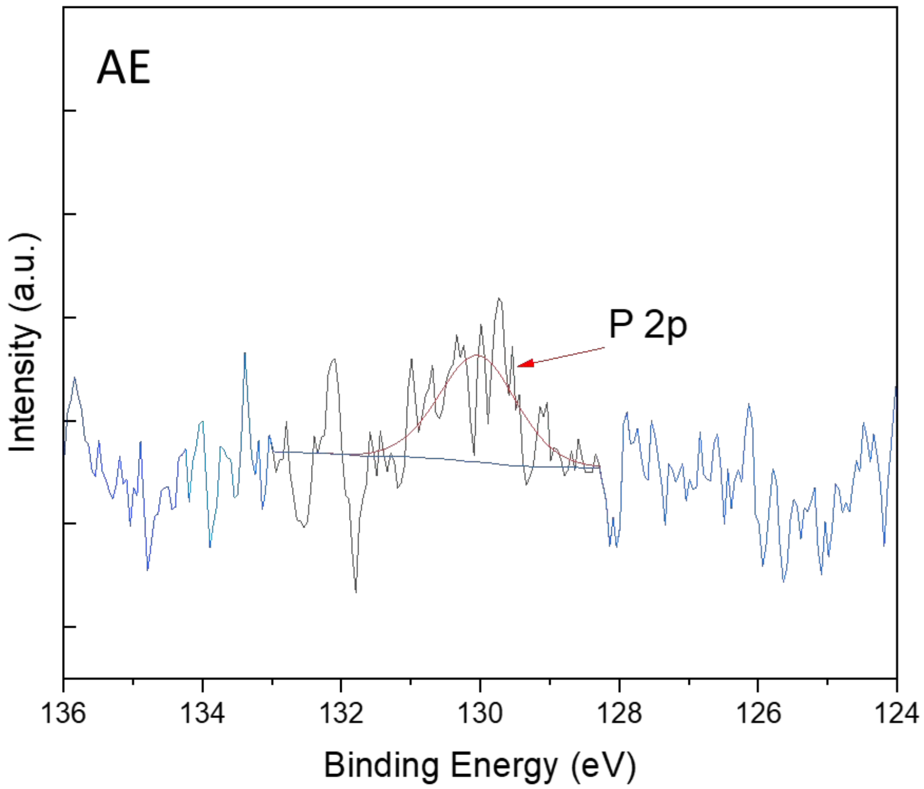

Figure S4. XPS characterization of Cu-P catalyst before and after CO2 electroreduction. High-resolution P 2p spectrum after electrolysis demonstrating reduced but persistent phosphorus signals, indicating retained P-doped structure rather than selective leaching.

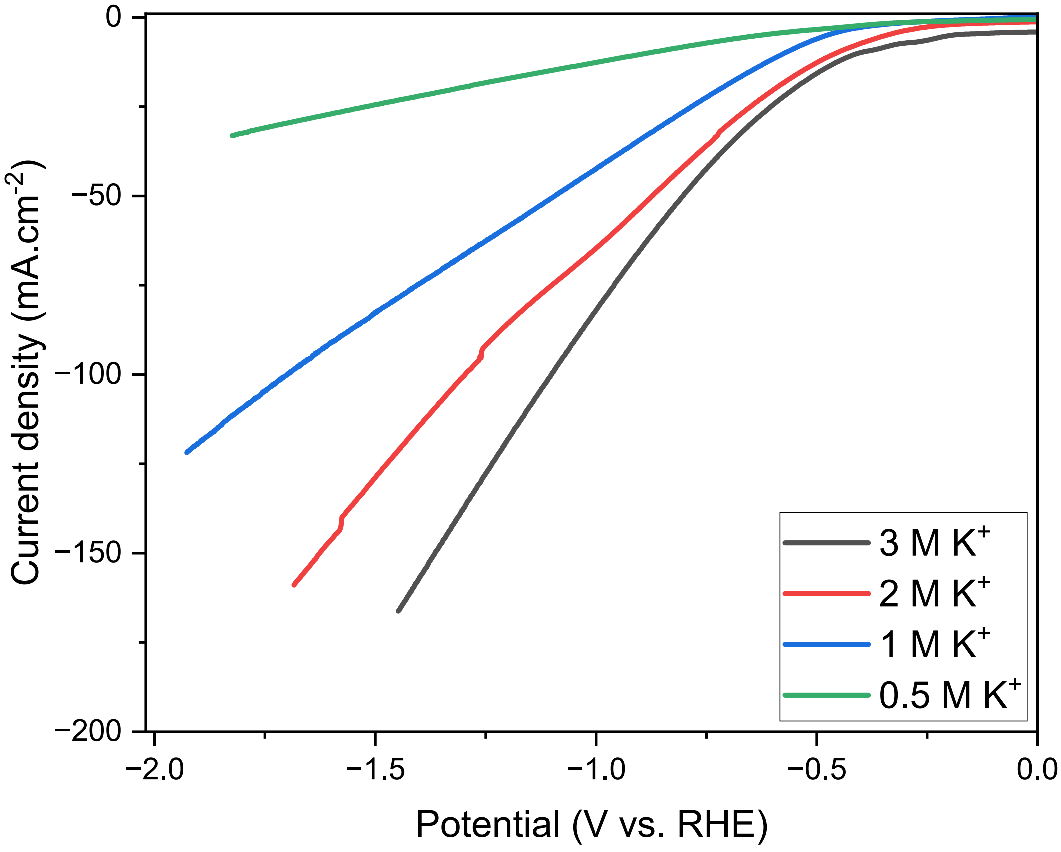

Figure S5: CO_2RR LSV curves of Cu-P showing the effect of K^+ concentration on catalyst performance in 0.5 M to 3.0 M KOH.

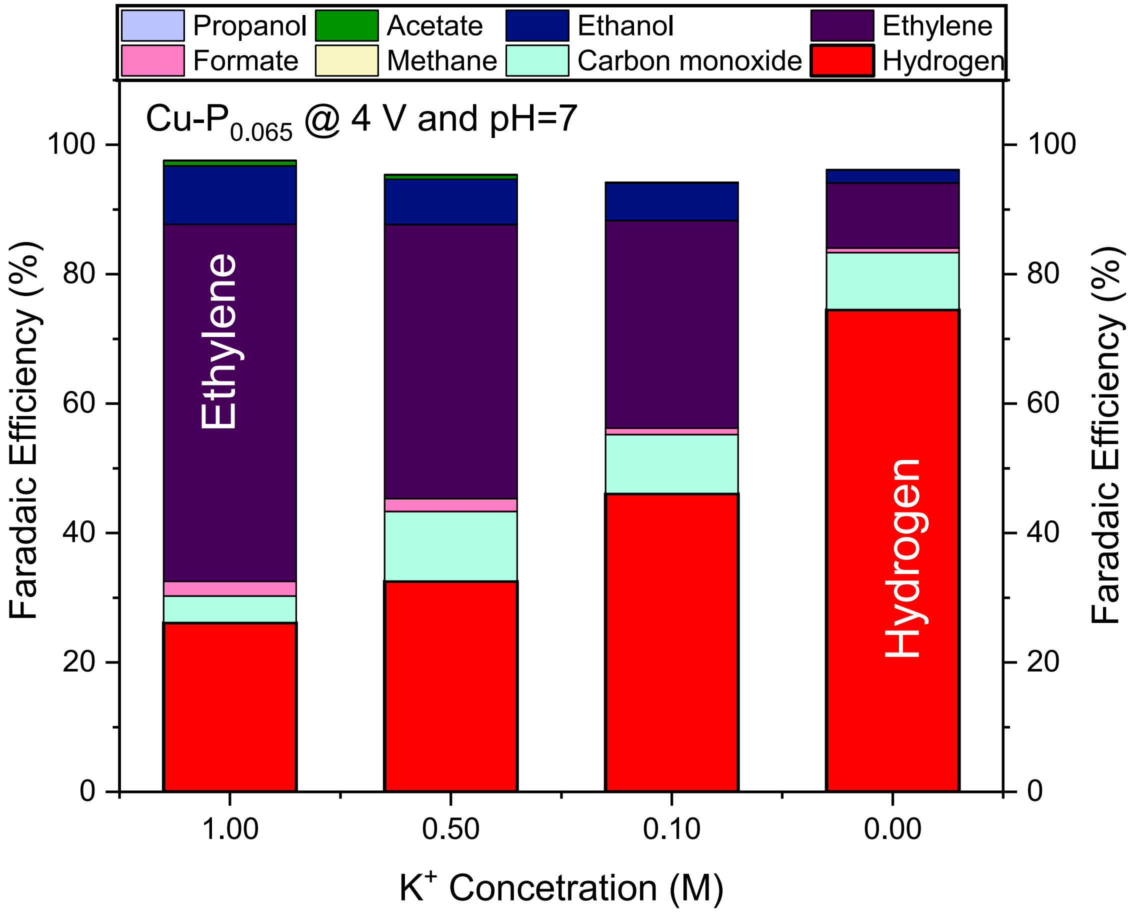

Figure S6. Effect of K+ concentration (0.1-2M) on product distribution at pH 6

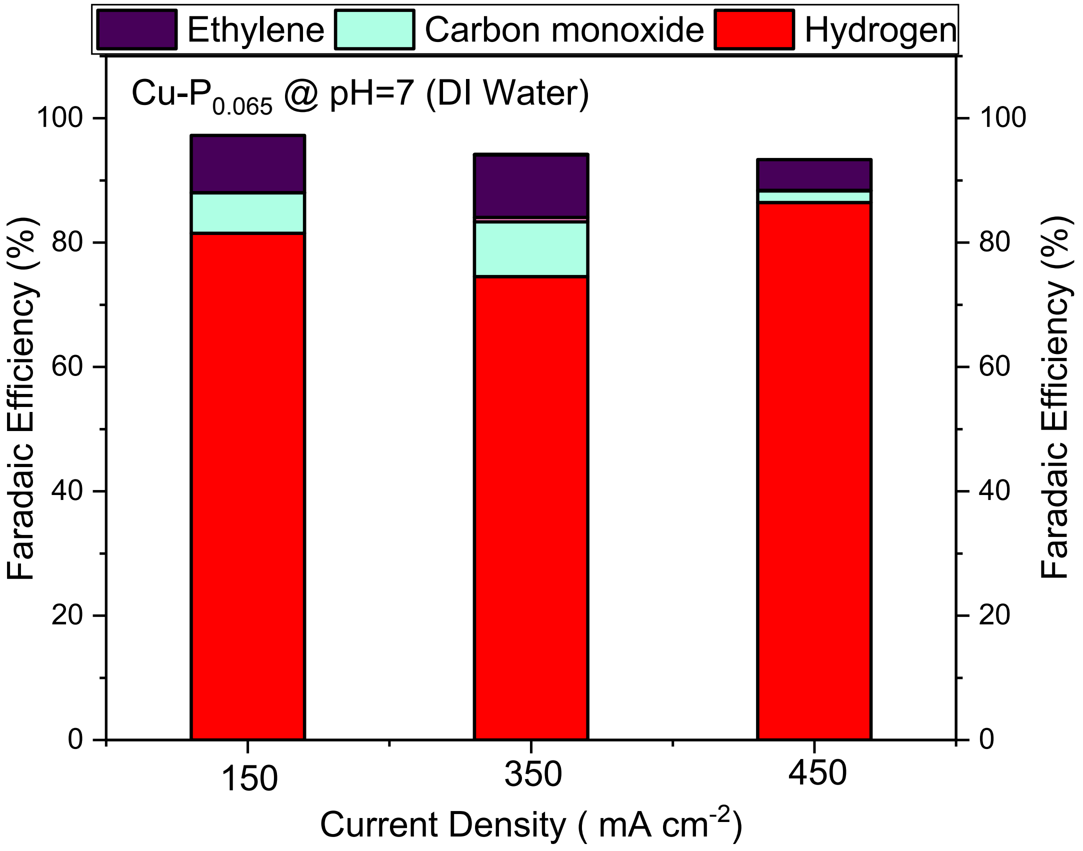

Figure S7. Faradaic Efficiency (%) versus Current Density (mA cm^{-2}) for Cu-$P_{0.065}$ at pH=7 in DI water.

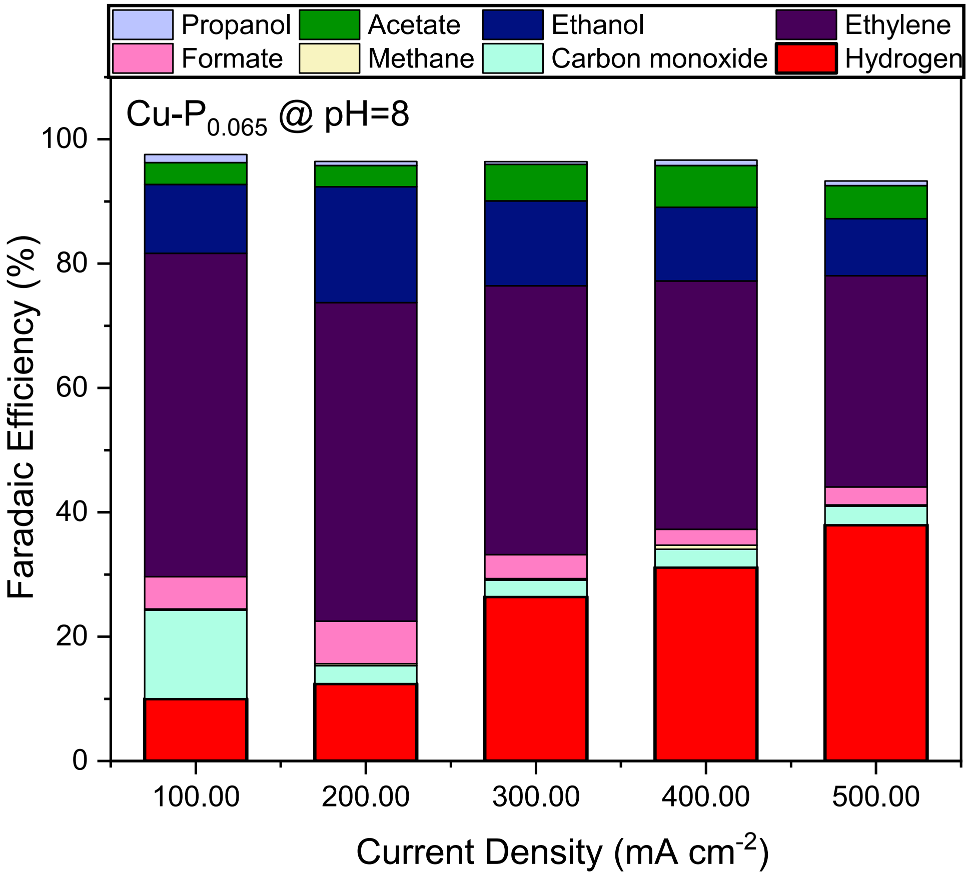

Figure S8. FEdistribution for CO_2 reduction products as a function of current density (100-500 mA cm^{-2}) at pH 14 using Cu-$P_{0.065}$ electrocatalyst in AEM system.

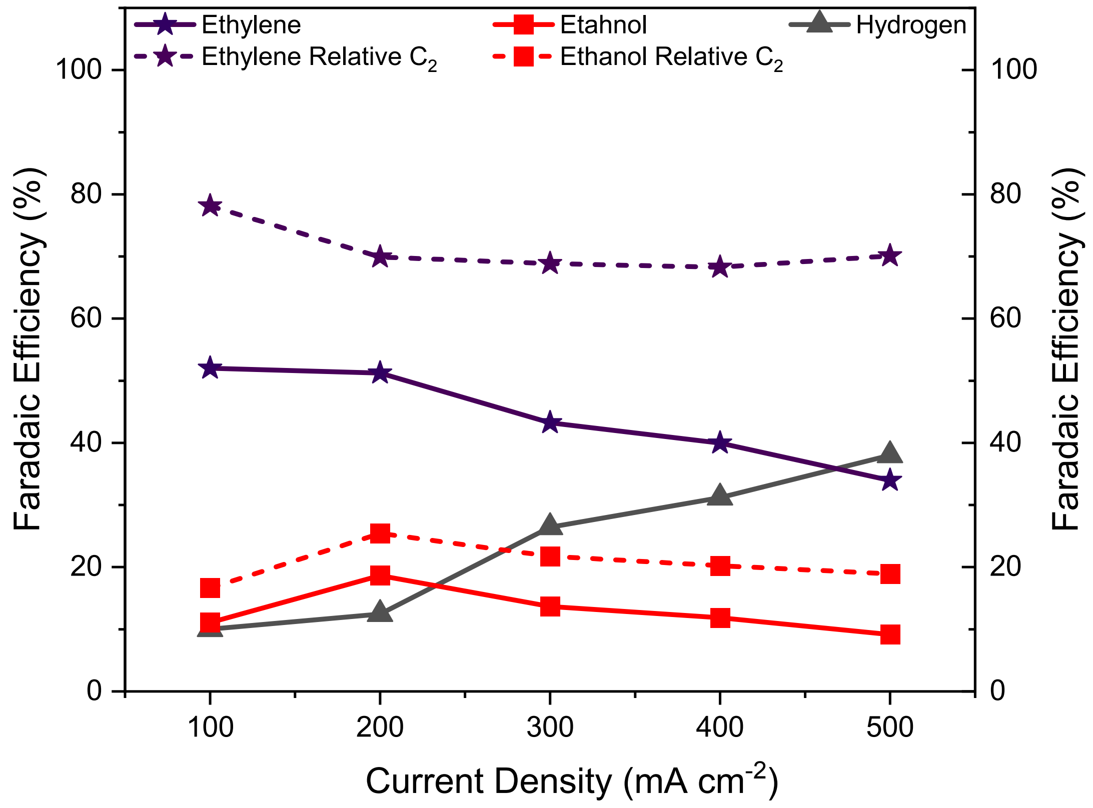

Figure S9. Relative distribution of C_2 products (ethylene and ethanol) across different current densities (100-500 mA cm^{-2}) at pH 8 using Cu-$P_{0.065}$ electrocatalyst.

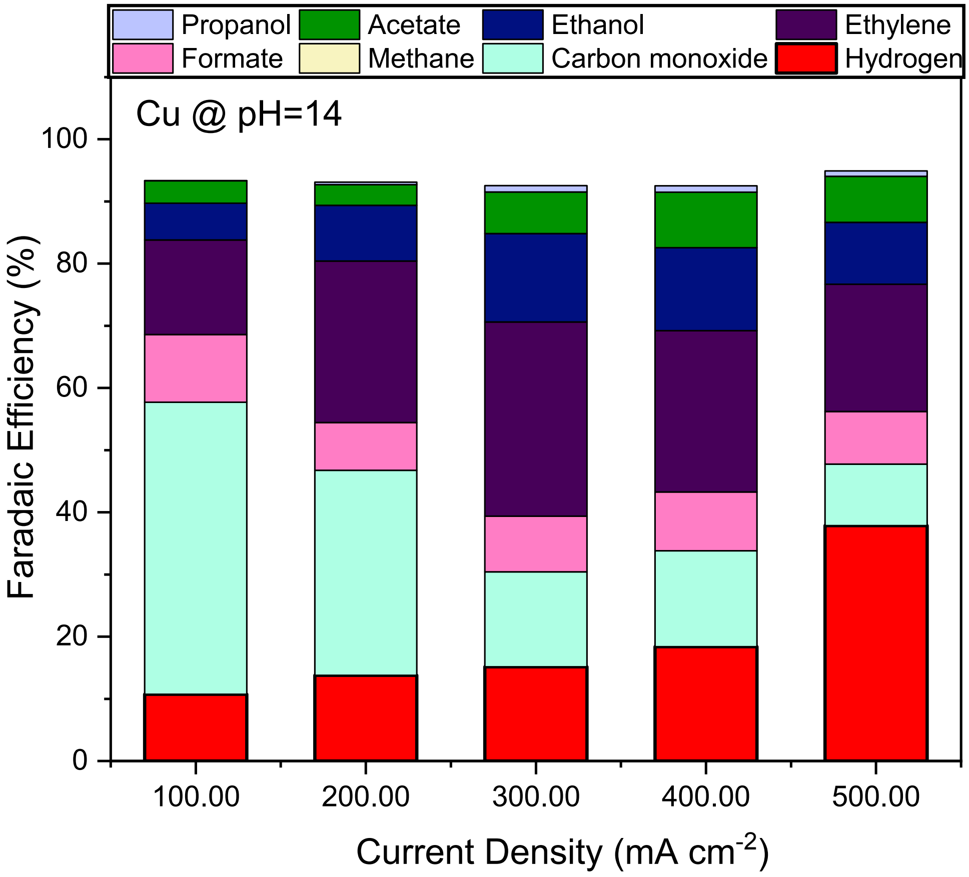

Figure S10. FEdistribution for CO_2 reduction products as a function of current density (100-500 mA cm^{-2}) at pH 14 using Cu-$P_{0.065}$ electrocatalyst in AEM system.

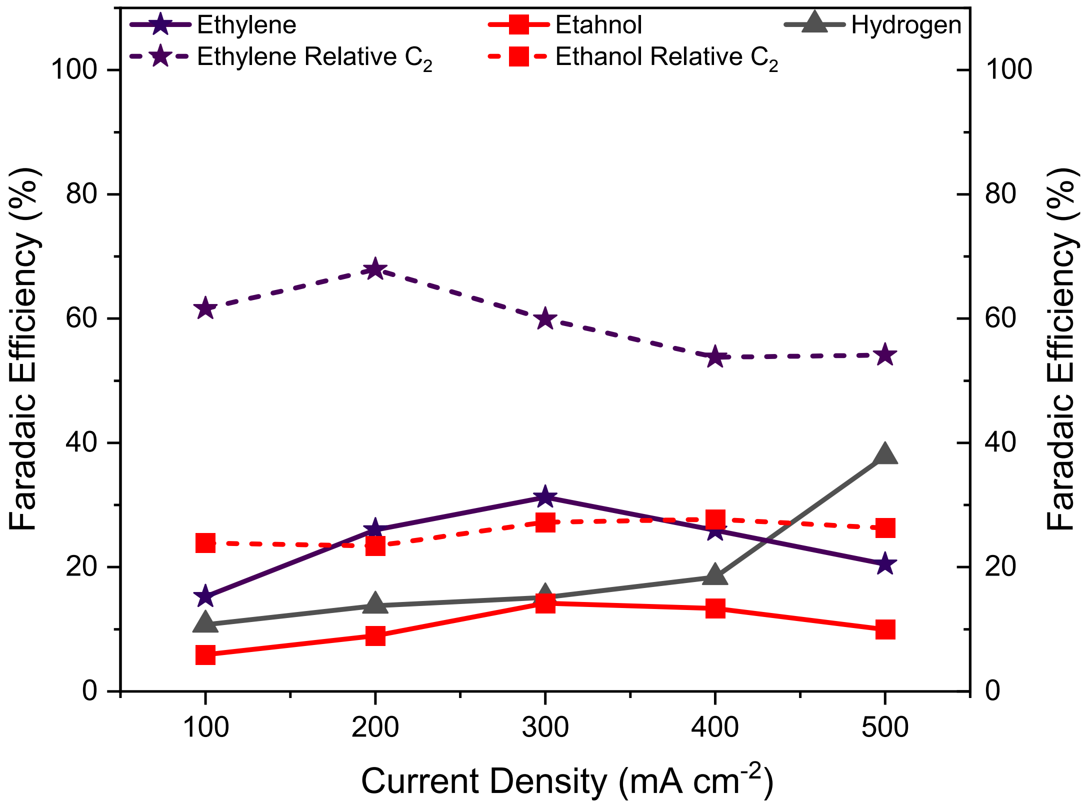

Figure S11 Relative distribution of C_2 products (ethylene and ethanol) across different current densities (100-500 mA cm^{-2}) at pH 14 using Cu-$P_{0.065}$ electrocatalyst.

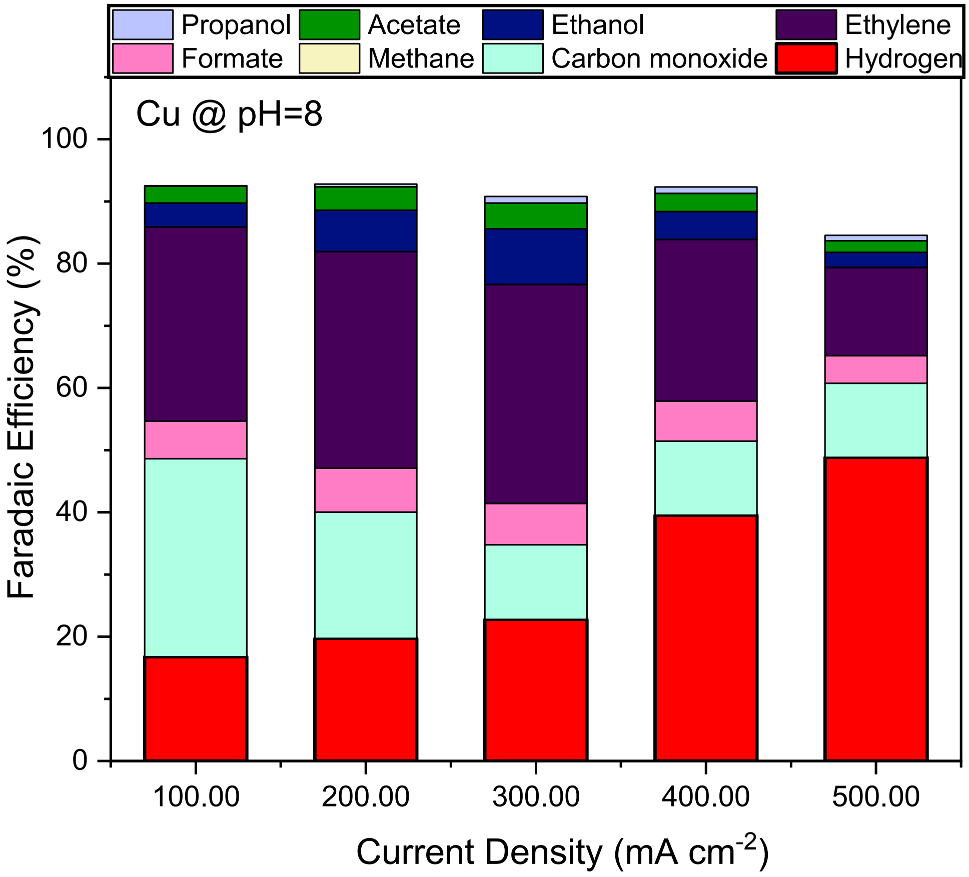

Figure S12. FEdistribution for CO_2 reduction products as a function of current density (100-500 mA cm^{-2}) at pH 8 using Cu electrocatalyst in AEM system.

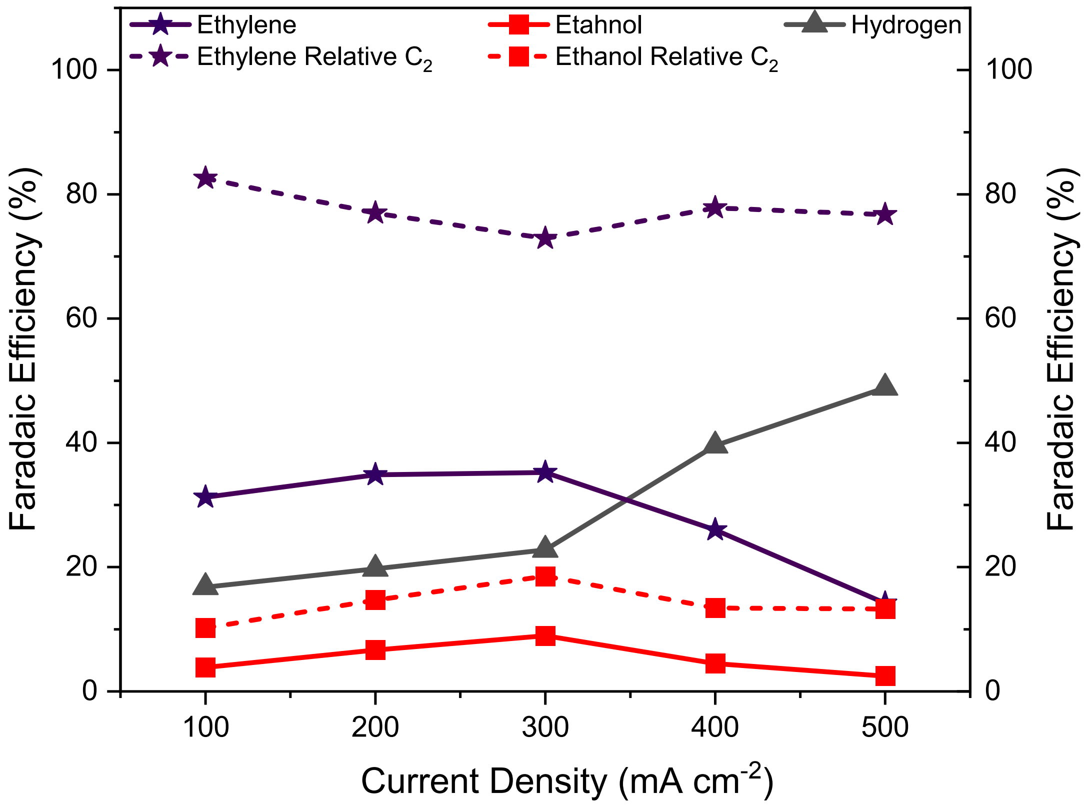

Figure S13. Relative distribution of C_2 products (ethylene and ethanol) across different current densities (100-500 mA cm^{-2}) at pH 6 using Cu electrocatalyst.

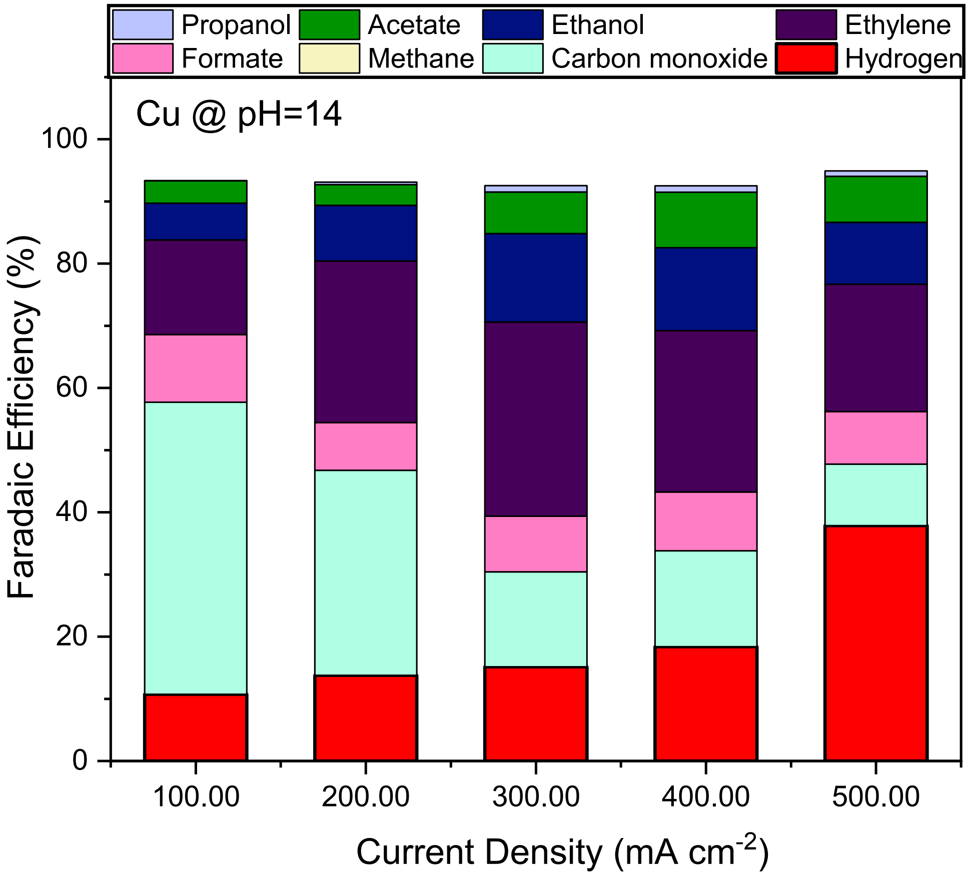

Figure S14. FEdistribution for CO_2 reduction products as a function of current density (100-500 mA cm^{-2}) at pH 14 using Cu electrocatalyst in AEM system.

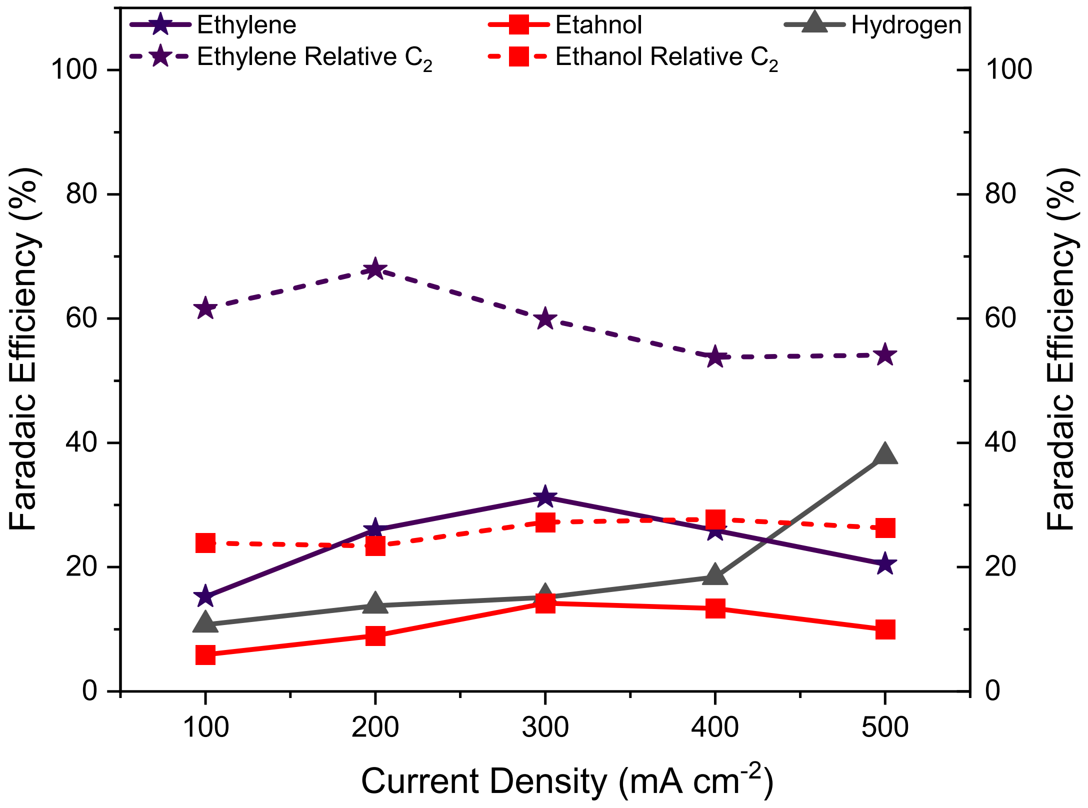

Figure S15. Relative distribution of C_2 products (ethylene and ethanol) across different current densities (100-500 mA cm^{-2}) at pH 14 using Cu electrocatalyst.

Table S2: Comparison to previous MEA electrolyzer for ethylene

Ref.	pH	FE C_2H_4 (%)	Current Density (mA cm^{-2})	Stability (h)
This work	6	72.84	300	500
This work	6	51.52	500	-
Lee et al [1]	14	54.5	281	6
Sang et al [2]	14	39.8	3.4	35
Adnan et al[3]	14	56	2.95	110
Dauda et al [4]	8	52	150	225
Kim et al [5]	8	62.5	150	180
Wu et al [6]	8	56	325	100
Li et al [7]	8	64	3.85	190
Wang et al [8]	8	60	3.7	65
Gabardo et al [9]	8	50	4.0	100
Xu el al [10]	8	56	3.8	236
Li et al [11]	7	48	308	5
zhang et al [12]	4	73	150	150
Xia et al [13]	2	40	500	4.5
Ma et al [14]	1	43	560	30

Supporting Note: Density functional theory calculations

Periodic plane wave density functional theory (DFT) computations were carried out utilizing the Vienna ab initio simulation package (VASP)[15, 16] as integrated into the computational catalysis interface (CCI).[17] Plane waves with an energy cutoff of 396 eV were used together with the projector augmented wave (PAW) method[18, 19] to expand the electronic wave functions. A fast Fourier transform (FFT) grid with a cutoff equal to 1.5 times the plane wave cutoff was used for the real space representation of the wave functions. Exchange and correlation energies were described using the generalized gradient approximation (GGA) in the form of the Bayesian error estimation functional (BEEF-vdW).[20] Wave functions were converged until electronic energies varied less than 10^{-4} eV and structures were geometrically optimized until the forces on all atoms were less than 0.05 eV-Å^{-1}.

The nudged elastic band (NEB) method in conjunction with the dimer method was used to determine the transition states.[21-23] Due to its higher computational cost, the NEB method was initially used to roughly converge the minimum energy pathway between the reactant and product states to obtain an initial guess for the transition state. The dimer method was then used to refine this initial estimate to the final convergence criterion.

The Cu(100) surface was modeled as 4×4 close-packed periodic lattices with four atomic layers in the orthogonal direction and a 30 Å vacuum between slabs, with the bottom two layers fixed in their bulk positions. Surface calculations were performed using a constant potential (grand canonical) formalism as implemented in the VASPsol++ implicit electrolyte model.[24] The reference Fermi level $\mu_{\mathrm{e}^-}^{\mathrm{ref}} = \mu_{\mathrm{e}^-}^{\mathrm{SHE}} - eU_{\mathrm{SHE}}^{\mathrm{ref}}$ was set to −3.07 eV corresponding to a potential of −1.5 V vs SHE, where a value of $\mu_{\mathrm{e}^-}^{\mathrm{SHE}} = -4.57$ eV is used for the Fermi level of the standard hydrogen electrode as obtained by the procedure in ref.[24] Total Landau free energies were

computed by adding zero-point vibrational energy (E_{ZPVE}) and a contribution from the harmonic vibrational partition function (A_{vib}, computed at 298 K) to the electronic Landau free energy (Ω_{el}) obtained from the VASP/VASPsol++ calculations,

$$\Omega^{\circ} = \Omega_{\mathrm{el}} + E_{\mathrm{ZPVE}} + A_{\mathrm{vib}} - n_{\mathrm{W}}\mu_{\mathrm{W}} \tag{S1}$$

These two contributions are computed from the vibrational frequencies obtained by a finite displacement phonon calculation in VASP/VASPsol++ using a displacement of 0.015 Å. To correct for the breakdown of the harmonic approximation with low-frequency modes, all vibrational modes below 50 cm^{-1} were set to this value. The final term in eq S1 accounts for the n_{W} explicit water molecules that hydrogen bond to a given intermediate or transition state on the surface. These are included using a hybrid implicit+explicit solvation framework to account for the absence of hydrogen bonding in purely implicit solvation models; however, they are excluded from the phonon calculations since the harmonic approximation is not appropriate for describing the flexibility of hydrogen bonds. In order to account for addition or removal of explicit waters when computing free energy differences, we specify a chemical potential μ_{W} that is determined by the condition that a molecule of water is solvated to the same extent by four explicit waters within a hybrid solvation model as it is by implicit water alone. This leads to a value of $\mu_{\mathrm{W}} = \frac{1}{4}\left(\Omega^{\circ}_{\mathrm{H_2O\cdot 4W}} - \Omega^{\circ}_{\mathrm{H_2O}}\right)$, where the free energies of solvated water in the implicit+explicit ($\Omega^{\circ}_{\mathrm{H_2O\cdot 4W}}$) and purely implicit ($\Omega^{\circ}_{\mathrm{H_2O}}$) models are calculated by eq S2 discussed below.

Solvated species were modeled within a 16×16×16 Å^3 unit cell at constant charge (rather than constant potential). In addition to the vibrational contributions, we also add contributions to

the free energy from the translational and rotational partition functions ($A^{\circ}_{\mathrm{trans}}$ and A_{rot}) computed using the ideal gas and rigid rotor approximations at 298 K. The free energy is then computed by,

(S2)

$$\Omega^{\circ} = \Omega_{\mathrm{el}} + E_{\mathrm{ZPVE}} + A^{\circ}_{\mathrm{trans}} + A_{\mathrm{rot}} + A_{\mathrm{vib}} - n_{\mathrm{W}}\mu_{\mathrm{W}}$$

with the translational free energy determined for a standard state concentration of 1 mol/L. Gas phase species are modeled in a similar way except without the implicit electrolyte, with the free energy computed by,

(S3)

$$\Omega^{\circ} = \Omega_{\mathrm{el}} + E_{\mathrm{ZPVE}} + A^{\circ}_{\mathrm{trans}} + A_{\mathrm{rot}} + A_{\mathrm{vib}} + k_{\mathrm{B}}T$$

The translational free energy is determined at a standard state pressure of 1 bar, and the additional $k_{\mathrm{B}}T$ term accounts for the PV term appearing in the free energy of an ideal gas at constant pressure.

Reaction and activation free energies

The reaction free energy for the Volmer step, whereby a proton donor HB transfers a proton to the surface, is computed at an electrode potential of $U^{\mathrm{ref}}_{\mathrm{SHE}}$ according to,

(S4)

$$\Delta\Omega^{\circ}_{\mathrm{ref}} = \Omega^{\circ}_{\mathrm{H^*}} - \Omega^{\circ}_{\mathrm{surf}} + \Omega^{\circ}_{\mathrm{B}} - \Omega^{\circ}_{\mathrm{HB}}$$

The free energies $\Omega^{\circ}_{\mathrm{surf}}$ and $\Omega^{\circ}_{\mathrm{H^*}}$ are computed for the clean Cu(100) surface with and without an adsorbed H atom, respectively, while $\Omega^{\circ}_{\mathrm{HB}}$ and $\Omega^{\circ}_{\mathrm{B}}$ are computed for the proton donor HB and its conjugate base B in the electrolyte. Similarly, the activation free energy is computed as,

(S5)

$$\Delta\Omega^{\ddagger}_{\mathrm{ref}} = \Omega^{\circ}_{\mathrm{TS}} - \Omega^{\circ}_{\mathrm{surf}} - \Omega^{\circ}_{\mathrm{HB}}$$

where $\Omega^{\circ}_{\mathrm{TS}}$ corresponds to the transition state for the Volmer step with proton donor HB.

The effect of pH and the pK_a of the proton donor on the reaction free energy can be accounted for by first writing the Landau free energies as Gibbs free energies ($\Omega \equiv G - q\mu^{\mathrm{ref}}_{\mathrm{e}^-}$),

(S6)

$$\Delta\Omega^{\circ} = G^{\circ}_{\mathrm{H}^*} - G_{\mathrm{surf}} + G^{\circ}_{\mathrm{B}} - G^{\circ}_{\mathrm{HB}} - \Delta q\mu_{\mathrm{e}^-}$$

where Δq is the change in the charge q of the explicit system during the reaction, using an "electron is postive" convention. Since the change in surface charge associated with H adsorption is almost negligible, we assume that $\Delta q \approx q_{\mathrm{B}} - q_{\mathrm{HB}} = 1$. This can be written as a function of pH and the proton donor pK_a using the following definitions,

(S7)

$$k_{\mathrm{B}}T \ln 10 \times \mathrm{p}K_{\mathrm{a}} \equiv G^{\circ}_{\mathrm{B}} - G^{\circ}_{\mathrm{HB}} + \mu^{\circ}_{\mathrm{H}^+}$$

$$k_{\mathrm{B}}T \ln 10 \times \mathrm{pH} \equiv \mu^{\circ}_{\mathrm{H}^+} - \mu_{\mathrm{H}^+}$$

where $\mu^{\circ}_{\mathrm{H}^+}$ is the proton chemical potential at a pH of zero and μ_{H^+} is the value at the acutal pH. This leads to the following expression for the reaction free energy as a function of pH and proton donor pK_a,

(S8)

$$\Delta\Omega^{\circ}(\mathrm{p}K_{\mathrm{a}},\mathrm{pH}) = \Delta\Omega^{\circ}_{\mathrm{ref}} + k_{\mathrm{B}}T \ln 10 \times (\mathrm{p}K_{\mathrm{a}} - \mathrm{pH})$$

where we have used the computational hydrogen electrode expressed as,

(S9)

$$\mu_{e^-} + \mu_{H^+} = \frac{1}{2} G^\circ_{H_{2(g)}} - eU_{RHE}$$

to rewrite the reaction free energy at the reference condition where $\mathrm{pH} = \mathrm{p}K_a$ and $U_{SHE} = U^{ref}_{SHE}$,

(S10)

$$\Delta\Omega^\circ_{ref} = G^\circ_{H^*} - G_{surf} - \frac{1}{2} G^\circ_{H_{2(g)}} + eU_{RHE}$$

The potential with respect to the reversible hydrogen electrode is defined as,

(S11)

$$U_{RHE} \equiv U_{SHE} + k_B T \ln 10 \times \mathrm{Ph}$$

and is held constant as the pH is varied so that $\Delta\Omega^\circ_{ref}$ is independent of pH.

To approximate the effect of pH and proton donor pK_a on the activation free energy, we express it in terms of the reaction free energy using the connection between transition state theory and Marcus theory that we have employed in previous work,[25]

(S12)

$$\Delta\Omega^\ddagger(\mathrm{p}K_a,\mathrm{pH}) = \frac{\left(\Delta\Omega^\circ(\mathrm{p}K_a,\mathrm{pH}) - \beta_R + \beta_P + \lambda(\mathrm{p}K_a)\right)^2}{4\lambda(\mathrm{p}K_a)} + \beta_R$$

The energetic quantities β_R and β_P account for the fact that the Marcus theory relationship should be based on the precursor states R and P for the forward and reverse reactions, respectively, rather than the initial and final states used to define the reaction free energy. These states are defined as

the pair of local minima that are directly connected by the transition state so that the transition state will fall into state R if displaced in the reverse direction or state P if displaced in the forward direction. In state R, the proton donor is sitting on the surface with the proton pointing towards the active site as depicted in **Figure S16** below for HB = H_2O. The quantity β_R accounts for the free energy cost associated with moving the proton donor from the bulk electrolyte (at the standard concentration of 1 mol/L) to the reactant precursor state R,

$$\beta_\mathrm{R} = \Omega_\mathrm{R}^\circ - \Omega_\mathrm{surf}^\circ - G_\mathrm{HB}^\circ \tag{S13}$$

Likewise, state P has the deprotonated form of the proton donor (hydroxide in the case of HB = H_2O) sitting near the adsorbed H atom as depicted in **Figure S16** below. The quantity β_P accounts for the free energy cost associated with moving the deprotonated proton donor B from the bulk electrolyte (again, at the standard state concentration of 1 mol/L) to the product precursor state P,

$$\beta_\mathrm{P} = \Omega_\mathrm{P}^\circ - \Omega_\mathrm{H^*}^\circ - G_\mathrm{B}^\circ \tag{S14}$$

Finally, we assume that the reorganization energy λ has a linear dependence on the proton donor pK_a, being lower for a more acidic donor,

$$\lambda(\mathrm{p}K_\mathrm{a}) = \lambda_\mathrm{ref} + \alpha k_\mathrm{B} T \ln 10 \times \left(\mathrm{p}K_\mathrm{a} - \mathrm{p}K_\mathrm{a}^\mathrm{ref}\right) \tag{S15}$$

where α is a positive constant. The quantity λ_{ref} is determined for a reference proton donor (HB = H2O in this case) with a pK_{a} equal to p$K_{\text{a}}^{\text{ref}}$ so that the activation free energy given by eq S12 matches the DFT value $\Delta\Omega_{\text{ref}}^{\ddagger}$ computed for the reference donor by eq S5, both with pH = p$K_{\text{a}}^{\text{ref}}$,

(S16)

$$\Delta\Omega_{\text{ref}}^{\ddagger} = \frac{(\Delta\Omega_{\text{ref}} - \beta_{\text{R}} + \beta_{\text{P}} + \lambda_{\text{ref}})^2}{4\lambda_{\text{ref}}} + \beta_{\text{R}}$$

Volmer step on Cu(100) with water as the proton donor

To determine the values of $\Delta\Omega_{\text{ref}}^{\circ}$, λ_{ref}, β_{R}, and β_{P} in eqs 10, 12, and 15, DFT calculations were performed for the Volmer step on Cu(100) with water acting as the proton donor so that HB = H_2O and B = OH^-. The structures of the transition state as well as the precursor states R and P are depicted in **Figure S16**. In all three states, the oxygen of water is hydrogen bonded to three explicit waters. When computing the free energies of H_2O and OH^- in the electrolyte, both are hydrogen bonded to four explicit waters as in ref.[24]

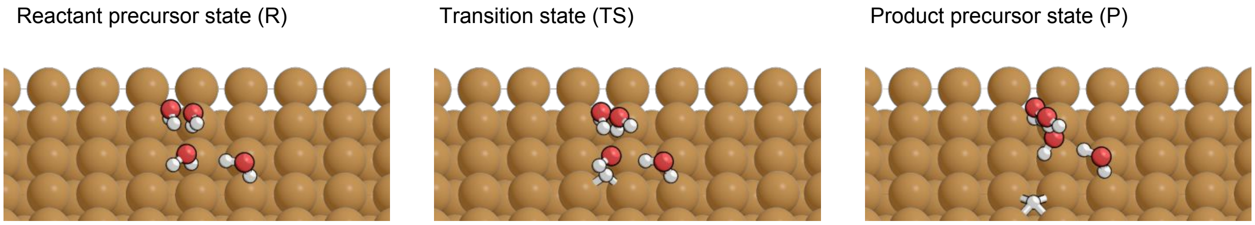

Figure S16. Optimized structures for the reacant precursor state, the transition state, and the product precursor state.

The results from the DFT calculations are reported in **Table S3**. Using these results, we obtain values of $\Delta\Omega^{\circ}_{\mathrm{ref}}$, $\Delta\Omega^{\ddagger}_{\mathrm{ref}}$, λ_{ref}, β_{R}, and β_{P} reported in **Table S4**. A value of $\alpha = 0.4$ was assumed for **eq S15**.

Table S3. Results of VASP/VASPsol++ calculations. All energies are in eV.

	n_{W}	Ω_{el}	E_{ZPVE}	A_{vib}	$A^{\circ}_{\mathrm{trans}} + A_{\mathrm{rot}}$
$H_{2(g)}$		-7.13	0.27	0.00	-0.31
$H_2O_{(aq)}$		-13.12	0.57	0.00	-0.40
H_2O	4	-66.04	0.57	0.00	-0.40
OH^-	4	-65.69	0.24	0.00	-0.36
surface		-23.21	0.48	-0.78	
R	3	-76.10	1.10	-0.92	
TS	3	-75.16	0.86	-0.98	
P	3	-75.79	0.85	-0.97	
H*		-26.79	0.55	-0.87	

Table S4. Parameters for the kinetic model of the Volmer step in eV.

$\Delta\Omega^{\circ}_{\mathrm{ref}}$	$\Delta\Omega^{\ddagger}_{\mathrm{ref}}$	λ_{ref}	β_{R}	β_{P}
-0.46	0.88	2.52	0.24	0.72

Kinetic model of the Volmer step with different proton donors

The total rate of the Volmer step is given by a sum over the rates of the individual proton donors,

$$r_{\mathrm{tot}} = \sum_i k(\mathrm{p}K_{\mathrm{a},i},\mathrm{pH})C_i(\mathrm{pH}) \tag{S17}$$

where the ith proton donor has a $\mathrm{p}K_{\mathrm{a}}$ equal to $\mathrm{p}K_{\mathrm{a},i}$. The rate coefficient k for each proton donor is computed from the corresponding activation free energy using transition state theory,

(S18)

$$k(\mathrm{p}K_{\mathrm{a},i},\mathrm{pH}) = \frac{k_{\mathrm{B}}T}{h}\exp\left(-\frac{\Delta\Omega^{\ddagger}(\mathrm{p}K_{\mathrm{a},i},\mathrm{pH})}{k_{\mathrm{B}}T}\right)$$

We consider seven proton donors in the model: two based on water (H_3O^+ and H_2O), two based on carbonic acid (H_2CO_3 and HCO_3^-), and three based on phosphoric acid (H_3PO_4, $H_2PO_4^-$, and HPO_4^{2-}). The pK_a values of these species are given in **Table S5**.

Table S5. pK_a values for the proton donors used in the kinetic model

H_3PO_4	2.14	H_2CO_3	3.75	H_3O^+	−1.74
$H_2PO_4^-$	7.20	HCO_3^-	10.33	H_2O	15.74
HPO_4^{2-}	12.37				

The concentration C_i must be specified in the rate expression for each proton donor as a function of pH. The concentrations of H_3O^+ and H_2O are given by,

(S19)

$$C_{\mathrm{H_3O^+}} = [\mathrm{H^+}]$$

$$C_{\mathrm{H_2O}} = [\mathrm{W}]$$

where $\log[\mathrm{H^+}] = -\,\mathrm{pH}$ is the proton concentration $[\mathrm{W}] = 55.5\ \mathrm{mol/L}$ is the concentration of liquid water. The concentrations of H_3PO_4, $H_2PO_4^-$, and HPO_4^{2-} are given as functions of pH by,

(S20)

$$C_{\mathrm{H_3PO_4}} = \frac{[\mathrm{H^+}]^3}{[\mathrm{H^+}]^3 + K_{\mathrm{a1}}[\mathrm{H^+}]^2 + K_{\mathrm{a1}}K_{\mathrm{a2}}[\mathrm{H^+}] + K_{\mathrm{a1}}K_{\mathrm{a2}}K_{\mathrm{a3}}}[\mathrm{P_{tot}}]$$

$$C_{\mathrm{H_2PO_4^-}} = \frac{K_{\mathrm{a1}}[\mathrm{H^+}]^2}{[\mathrm{H^+}]^3 + K_{\mathrm{a1}}[\mathrm{H^+}]^2 + K_{\mathrm{a1}}K_{\mathrm{a2}}[\mathrm{H^+}] + K_{\mathrm{a1}}K_{\mathrm{a2}}K_{\mathrm{a3}}}[\mathrm{P_{tot}}]$$

$$C_{\mathrm{HPO_4^{2-}}} = \frac{K_{\mathrm{a1}}K_{\mathrm{a2}}[\mathrm{H^+}]}{[\mathrm{H^+}]^3 + K_{\mathrm{a1}}[\mathrm{H^+}]^2 + K_{\mathrm{a1}}K_{\mathrm{a2}}[\mathrm{H^+}] + K_{\mathrm{a1}}K_{\mathrm{a2}}K_{\mathrm{a3}}}[\mathrm{P_{tot}}]$$

where $[\mathrm{P_{tot}}] =$ 1 mol/L is the total concentration of dissolved phosphate species and the acid dissociations constants K_{a1}, K_{a2}, and K_{a3} (given by $\log K_{\mathrm{a}} = -\,\mathrm{p}K_{\mathrm{a}}$) correspond to p$K_{\mathrm{a}}$ values of H_3PO_4, $H_2PO_4^-$, and HPO_4^{2-}, respectively.

Finally, the concentrations of H_2CO_3 and HCO_3^- are given by,

(S21)

$$C_{\mathrm{H_2CO_3}} = \frac{[\mathrm{H^+}]^2}{[\mathrm{H^+}]^2 + K_{\mathrm{a1}}[\mathrm{H^+}] + K_{\mathrm{a1}}K_{\mathrm{a2}}}[\mathrm{C_{tot}}]$$

$$C_{\mathrm{HCO_3^-}} = \frac{K_{\mathrm{a1}}[\mathrm{H^+}]}{[\mathrm{H^+}]^2 + K_{\mathrm{a1}}[\mathrm{H^+}] + K_{\mathrm{a1}}K_{\mathrm{a2}}}[\mathrm{C_{tot}}]$$

where K_{a1} and K_{a2} are the acid dissociation constants for H_2CO_3 and HCO_3^-, and $[\mathrm{C_{tot}}]$ is the concentration of total dissolved carbon. The latter is given by,

(S22)

$$[\mathrm{C_{tot}}] = \min\left([\mathrm{C_{tot}^0}],[\mathrm{C_{tot}^{max}}]\right)$$

$$\left[\mathrm{C_{tot}^{max}}\right] = K_{\mathrm{h}}P_{\mathrm{CO_2}}\left(1 + K_{\mathrm{a1}}[\mathrm{H^+}]^{-1} + K_{\mathrm{a1}}K_{\mathrm{a2}}[\mathrm{H^+}]^{-2}\right)$$

where $\left[\mathrm{C_{tot}^0}\right] =$ 1 mol/L is the nominal concentration of total dissolved carbon and $\left[\mathrm{C_{tot}^{max}}\right]$ is the maximum concentration of dissolved carbon that can exist in equilibrium with a partial pressure $P_{\mathrm{CO_2}} =$ 1 atm of CO_2. This limit accounts for the fact that the electrolyte will evolve CO_2 at lower pH until $[\mathrm{C_{tot}}]$ drops down to $\left[\mathrm{C_{tot}^{max}}\right]$, with $K_{\mathrm{h}} =$ 5.27×10^{-5} mol/L-atm describing the equilibrium between carbonic acid and gas phase CO_2 according to $[\mathrm{H_2CO_3}] = K_{\mathrm{h}}P_{\mathrm{CO_2}}$.

References

(1) Lee, W. H.; Lim, C.; Lee, S. Y.; Chae, K. H.; Choi, C. H.; Lee, U.; Min, B. K.; Hwang, Y. J.; Oh, H.-S. Highly selective and stackable electrode design for gaseous CO2 electroreduction to ethylene in a zero-gap configuration. *Nano Energy* **2021**, *84*, 105859. DOI: https://doi.org/10.1016/j.nanoen.2021.105859.

(2) Sang, J.; Wei, P.; Liu, T.; Lv, H.; Ni, X.; Gao, D.; Zhang, J.; Li, H.; Zang, Y.; Yang, F.; et al. A Reconstructed Cu2P2O7 Catalyst for Selective CO2 Electroreduction to Multicarbon Products. *Angewandte Chemie International Edition* **2022**, *61* (5), e202114238. DOI: https://doi.org/10.1002/anie.202114238.

(3) Adnan, M. A.; Shayesteh Zeraati, A.; Nabil, S. K.; Al-Attas, T. A.; Kannimuthu, K.; Dinh, C.-T.; Gates, I. D.; Kibria, M. G. Directly-Deposited Ultrathin Solid Polymer Electrolyte for Enhanced CO2 Electrolysis. *Advanced Energy Materials* **2023**, *13* (12), 2203158. DOI: https://doi.org/10.1002/aenm.202203158.

(4) Dauda, M. O.; Hendershot, J.; Bello, M.; Park, J.; Loaiza Orduz, A.; Lombardo, N.; Kizilkaya, O.; Sprunger, P.; Engler, A.; Plaisance, C.; et al. Electrochemical Reduction of CO2: A Common Acetyl Path to Ethylene, Ethanol or Acetate. *J. Electrochem. Soc.* **2024**.

(5) Kim, J.-Y.; Hong, D.; Lee, J.-C.; Kim, H. G.; Lee, S.; Shin, S.; Kim, B.; Lee, H.; Kim, M.; Oh, J.; et al. Quasi-graphitic carbon shell-induced Cu confinement promotes electrocatalytic CO2 reduction toward C2+ products. *Nat Commun* **2021**, *12* (1), 3765. DOI: 10.1038/s41467-021-24105-9.

(6) Wu, H.; Li, J.; Qi, K.; Zhang, Y.; Petit, E.; Wang, W.; Flaud, V.; Onofrio, N.; Rebiere, B.; Huang, L.; et al. Improved electrochemical conversion of CO2 to multicarbon products by using molecular doping. *Nat Commun* **2021**, *12* (1), 7210. DOI: 10.1038/s41467-021-27456-5 (acccessed 2023/06/10/23:17:45). From www.nature.com.

(7) Li, F.; Thevenon, A.; Rosas-Hernández, A.; Wang, Z.; Li, Y.; Gabardo, C. M.; Ozden, A.; Dinh, C. T.; Li, J.; Wang, Y. Molecular tuning of CO2-to-ethylene conversion. *Nature* **2020**, *577* (7791), 509-513.

(8) Wang, Y.; Wang, Z.; Dinh, C.-T.; Li, J.; Ozden, A.; Golam Kibria, M.; Seifitokaldani, A.; Tan, C.-S.; Gabardo, C. M.; Luo, M.; et al. Catalyst synthesis under CO2 electroreduction favours faceting and promotes renewable fuels electrosynthesis. *Nat Catal* **2020**, *3* (2), 98-106. DOI: 10.1038/s41929-019-0397-1.

(9) Gabardo, C. M.; O'Brien, C. P.; Edwards, J. P.; McCallum, C.; Xu, Y.; Dinh, C.-T.; Li, J.; Sargent, E. H.; Sinton, D. Continuous Carbon Dioxide Electroreduction to Concentrated Multi-carbon Products Using a Membrane Electrode Assembly. *Joule* **2019**, *3* (11), 2777-2791. DOI: https://doi.org/10.1016/j.joule.2019.07.021.

(10) Xu, Y.; Edwards, J. P.; Liu, S.; Miao, R. K.; Huang, J. E.; Gabardo, C. M.; O'Brien, C. P.; Li, J.; Sargent, E. H.; Sinton, D. Self-Cleaning CO2 Reduction Systems: Unsteady Electrochemical Forcing Enables Stability. *ACS Energy Lett.* **2021**, *6* (2), 809-815. DOI: 10.1021/acsenergylett.0c02401.

(11) Li, W.; Yin, Z.; Gao, Z.; Wang, G.; Li, Z.; Wei, F.; Wei, X.; Peng, H.; Hu, X.; Xiao, L.; et al. Bifunctional ionomers for efficient co-electrolysis of CO2 and pure water towards ethylene production at industrial-scale current densities. *Nature Energy* **2022**, *7* (9), 835-843. DOI: 10.1038/s41560-022-01092-9.

(12) Zhang, J.; Guo, C.; Fang, S.; Zhao, X.; Li, L.; Jiang, H.; Liu, Z.; Fan, Z.; Xu, W.; Xiao, J.; et al. Accelerating electrochemical CO2 reduction to multi-carbon products via asymmetric intermediate binding at confined nanointerfaces. *Nat Commun* **2023**, *14* (1), 1298. DOI: 10.1038/s41467-023-36926-x.
(13) Xie, Y.; Ou, P.; Wang, X.; Xu, Z.; Li, Y. C.; Wang, Z.; Huang, J. E.; Wicks, J.; McCallum, C.; Wang, N.; et al. High carbon utilization in CO2 reduction to multi-carbon products in acidic media. *Nat Catal* **2022**, *5* (6), 564-570. DOI: 10.1038/s41929-022-00788-1.
(14) Ma, Z.; Yang, Z.; Lai, W.; Wang, Q.; Qiao, Y.; Tao, H.; Lian, C.; Liu, M.; Ma, C.; Pan, A.; et al. CO2 electroreduction to multicarbon products in strongly acidic electrolyte via synergistically modulating the local microenvironment. *Nat Commun* **2022**, *13* (1), 7596. DOI: 10.1038/s41467-022-35415-x.
(15) Kresse, G.; Furthmüller, J. Efficiency of ab-initio total energy calculations for metals and semiconductors using a plane-wave basis set. *Computational Materials Science* **1996**, *6* (1), 15-50. DOI: https://doi.org/10.1016/0927-0256(96)00008-0.
(16) Kresse, G.; Furthmüller, J. Efficient iterative schemes for ab initio total-energy calculations using a plane-wave basis set. *Phys Rev B Condens Matter* **1996**, *54* (16), 11169-11186. DOI: 10.1103/physrevb.54.11169 From NLM.
(17) Kravchenko, P.; Plaisance, C.; Hibbitts, D. A new computational interface for catalysis. **2019**.
(18) Blöchl, P. E. Projector augmented-wave method. *Physical review B* **1994**, *50* (24), 17953.
(19) Kresse, G.; Joubert, D. From ultrasoft pseudopotentials to the projector augmented-wave method. *Physical Review B* **1999**, *59* (3), 1758-1775. DOI: 10.1103/PhysRevB.59.1758.
(20) Wellendorff, J.; Lundgaard, K. T.; Møgelhøj, A.; Petzold, V.; Landis, D. D.; Nørskov, J. K.; Bligaard, T.; Jacobsen, K. W. Density functionals for surface science: Exchange-correlation model development with Bayesian error estimation. *Physical Review B—Condensed Matter and Materials Physics* **2012**, *85* (23), 235149.
(21) Henkelman, G.; Uberuaga, B. P.; Jónsson, H. A climbing image nudged elastic band method for finding saddle points and minimum energy paths. *The Journal of Chemical Physics* **2000**, *113* (22), 9901-9904. DOI: 10.1063/1.1329672 (acccessed 4/27/2025).
(22) Henkelman, G.; Jónsson, H. Improved tangent estimate in the nudged elastic band method for finding minimum energy paths and saddle points. *The Journal of Chemical Physics* **2000**, *113* (22), 9978-9985. DOI: 10.1063/1.1323224 (acccessed 4/27/2025).
(23) Henkelman, G.; Jónsson, H. A dimer method for finding saddle points on high dimensional potential surfaces using only first derivatives. *The Journal of Chemical Physics* **1999**, *111* (15), 7010-7022. DOI: 10.1063/1.480097 (acccessed 4/27/2025).
(24) Islam, S. M. R.; Khezeli, F.; Ringe, S.; Plaisance, C. An implicit electrolyte model for plane wave density functional theory exhibiting nonlinear response and a nonlocal cavity definition. *J Chem Phys* **2023**, *159* (23). DOI: 10.1063/5.0176308 From NLM.
(25) Khezeli, F.; Plaisance, C. Computational Design of an Electro-Organocatalyst for Conversion of CO2 into Formaldehyde. *The Journal of Physical Chemistry A* **2024**, *128* (9), 1576-1592. DOI: 10.1021/acs.jpca.3c07806.
